# Supplementary material for: Deep learning to predict rapid progression of Alzheimer’s disease from pooled clinical trials: A retrospective study
Source: PLOS Digit Health. 2024 Apr 10;3(4):e0000479. doi: 10.1371/journal.pdig.0000479 (PMC11006164; doi:10.1371/journal.pdig.0000479)
Supplement: S1 Appendix — (DOCX) [file pdig.0000479.s001.docx]

## S1 Appendix. Additional materials, methods, and results.

### Gaussian mixture model

We fit a Gaussian mixture model (GMM) to the distribution of the four labels (ADAS-Cog14, ADCS-ADL, MMSE, CDR-SB) to assess the latent clusters. The natural separation of the distribution calculated by GMM was then compared with our predefined 10% threshold (S1 Fig).

###

**Imputation strategies**

We tried several imputation strategies to deal with missing data in our baseline variables and longitudinal variables. The baseline variables include patient demographics, comorbidities, and other neurocognitive measurements. The comorbidities are binary variables, and we only included those with ≤ 7 missing values out of 1603 samples (missing rate ~ 0.44%), which is small enough to legitimately impute the missing values as 0 (no existence) since it is the major class for all comorbidities. Then, for the remaining numerical baseline variables, we used two imputation strategies: (1) simple imputation with mean values of each column, which assumes the missing to be MCAR; (2) multiple imputation by chained equation (MICE), which assumes the missing to be MAR. For longitudinal variables, the missing values are mainly certain observations for some time steps in each patient. We further compared three imputation methods: (1) last observation carried forward (LOCF), assuming that the longitudinal data has temporal stability as the cohorts we used are placebo arms of clinical trials; (2) linear imputation, which assumes temporal linearity between observed time steps; (3) MICE, which assumes the missing to be MAR and depend on other observed values. For longitudinal imputation (2) and (3), we carefully avoided temporal data leakage by imputing data within the observed periods according to the specific observation periods for each model. Note that when using MICE, we only generated one imputed dataset for each training fold, considering the computational cost that we had several training settings and used 10-fold cross-validation. Thus, we have 6 combinations of imputation methods between baseline and longitudinal variables. The 6 imputation strategies were evaluated by the internal split and 10-fold cross-validation. The AUROC and AUPRC plots are reported in the S2 Fig. We found that the two imputation strategies for baseline variables do not show significant differences, while the MICE imputation for longitudinal variables showed marginal improvement. As a result, we selected MICE as our imputation strategy for both baseline and longitudinal variables.

### Rapid progression prediction model

We developed a rapid progression prediction model using recurrent neural networks that take input of baseline conditions and longitudinal features and output the probability of being rapid progressors. We used the GRU,^1^ which is a gated mechanism widely used in recurrent neural networks with an update gate and a reset gate to control the flow of information from the previous hidden state and the current inputs.

Specifically, let $D\in\mathbb{R}^{d_{0}}$ be demographics and comorbidities (sex, race, BMI, APOE, comorbid illness, etc.), $V_{0}\in\mathbb{R}^{d_{1}}$ be baseline efficacy measures (cognitive and psychological scores, brain volumetry, and lab results), $V_{n}\in\mathbb{R}^{d_{1}}$ be the efficacy measures of the n-th observation, $\Delta V_{n}=V_{n}-V_{0}$ be the changes from baseline to the n-th observation, and $Y\in\{0, 1\}$ be binary labels (1 if rapid progressor; 0 otherwise). We concatenated $D$, $V_{0}$, and $\Delta V_{n}$ to form the longitudinal feature at the n-th observation $X_{n}=[{D;V}_{0};{\Delta V}_{n}]$. Suppose $f_{\theta}(\cdot)\to\mathbb{R}^{d}$ be an embedding layer that maps original features into $d$-dimensional embeddings and $\theta$ is the embedding parameter to learn. Here $f_{\theta}$consists of a linear layer, a batch normalization layer, a ReLU active function, and a dropout layer. The embedding for the $X_{n}$ is then $E_{n}=f_{\theta}(X_{n})\in\mathbb{R}^{d}$ and is fed into a GRU to get the outputs $K_{n}\in\mathbb{R}^{d}$. These outputs are treated as both keys and values for the following attention mechanism. The query of the attention mechanism is a learnable vector $Q\in\mathbb{R}^{d}$. The attention weight of the feature at n-th observation is thus $a_{n}=Q^{\top}K/\sqrt{d}\mathbb{\in R}$. By introducing the learnable query vector, the model would learn to assign larger weights to more important time steps which would provide more information and contribute more to the final prediction. The output of the attention mechanism is the weighted sum of $K_{n}$ for all observations $n=1,2,...,N$, which is thus $O=\sum_{n=1}^{N} a_{n}K_{n}$. A final multilayer perceptron (MLP) (consisting of one hidden linear layer followed by batch normalization, ReLU activation, and dropout, and one final linear projection to a scalar) is attached to $O$ and outputs a scalar $\hat{y}$ as the predicted logit. The binary cross entropy loss between the output logit $\hat{y}$ and the ground-truth label $y$ is calculated by $l\left( \hat{y},y \right)=-\left[ p\cdot y\cdot\log\sigma\left( y \right)+\left( 1-y \right)\cdot\log\left( 1-\sigma\left( \hat{y} \right) \right) \right],$ where $p$ is the positive weights and $\sigma(\cdot)$ is the sigmoid function. In our setting, $p=9$ since the ratio between positive and negative samples is approximately 9, which will make the loss act as if there are 9 times more positive samples.

**Model calibration**

We calibrated all the models by fitting the spline calibration^2^ on the model predictions from corresponding validation sets and the ground-truth labels. We then averaged the calibrated probabilities over the 10 folds to get the final predictions on the test sets and plotted the calibration curves (here we used 7 bins) for all the models (S4 Fig). We also reported the expected calibration errors (ECEs) and Brier scores (BSs) for all the curves. ECE and BS are metrics that evaluate model calibration. The ECE is calculated by averaging the bin-weighted absolute differences between accuracy and confidence:

$$\mathrm{ECE}=\sum_{m=1}^{M} \frac{|B_{m}|}{N}\left| \mathrm{acc}\left( B_{m} \right)-\mathrm{con}\left( B_{m} \right) \right|$$

where $N$ is the sample size, $M$ is the number of bins (here $M=7$), $\left| B_{m} \right|$ is the number of samples in the bin $m$, $\mathrm{acc}\left( B_{m} \right)$ is the accuracy within bin $m$, and $\mathrm{con}\left( B_{m} \right)$ is the confidence of bin $m$ (averaged probabilities predicted by the models). The BS is defined by the mean squared difference between the probabilities predicted by the models and the ground-truth binary labels:

$$\mathrm{BS}=\frac{1}{N}\sum_{n=1}^{N} \left( p_{n}-g_{n} \right)^{2}$$

where $N$ is the sample size, $p_{n}$ is the predicted probability of sample $n$, and $g_{n}$ is the ground-truth label of sample $n$. We can summarize that our models achieved good calibration results overall, while models trained by the label defined by MMSE have better calibration than other ones.

**Attention weight heatmaps**

We visualized the attention weights for all models as heatmaps, where yellow indicates high attention while blue indicates low attention. For a specific model trained on $N$ observations, the attention weights are $A=\left[ a_{1},a_{2},\ldots a_{N} \right]$, where $a_{n}$ is calculated as described above (in the “Rapid progression prediction model” section). S3 Fig shows the attention weights averaged over 10 folds for all the models, and some patterns can be summarized: (1) when predicting labels defined by ADAS-Cog14 and ADCS-ADL, later time point had higher importance for model prediction; (2) when predicting labels defined by MMSE, earlier time points contributed more to the model prediction; (3) when predicting labels defined by CDR-SB, the attention maps did not show common patterns across different observation periods. However, the clinical insights of these patterns are not clear, and further research could investigate these findings.

### SHAP interpretation

The SHAP^3^ values were computed from coalition game theory to explain the contribution of each feature to the prediction. Higher SHAP values of features indicate more impact on the model prediction. Since SHAP is an additive feature attribution method, we can use the sum of SHAP values to explain the overall contribution of a group of features. The SHAP value of each longitudinal feature was calculated by adding the SHAP values of the baseline condition and the longitudinal changes from baseline to evaluate the overall impact of the feature on model prediction.

### Patient progression visualization

We selected the two most common and important cognitive domains, the plasma levels of Aβ_1‑42_ and the volumetric measurements for each model, and reported the mean/standard deviations of the longitudinal changes in those features from baseline to Week 80. We focused on the divergence of the trajectories between predicted positives (i.e., patients who are predicted as RPs) and predicted negatives (i.e., patients who are predicted as non-RPs). We also showed the trajectories of ground-truth positives (i.e., patients who are pre-defined as RPs) and ground-truth negatives (i.e., patients who are pre-defined as non-RPs) for comparison. When showing the progression of patients, we used LOCF to impute those temporal missing values for the purpose of visualization.

## References

1 Cho K, van Merriënboer B, Bahdanau D, Bengio Y. On the Properties of Neural Machine Translation: Encoder--Decoder Approaches. In: Proceedings of SSST-8, Eighth Workshop on Syntax, Semantics and Structure in Statistical Translation. Doha, Qatar: Association for Computational Linguistics, 2014: 103–11.

2 Lucena B. Spline-Based Probability Calibration. arXiv [stat.ML]. 2018; published online Sept 20. http://arxiv.org/abs/1809.07751.

3 Lundberg SM, Lee S-I. A unified approach to interpreting model predictions. In: Proceedings of the 31st International Conference on Neural Information Processing Systems. Red Hook, NY, USA: Curran Associates Inc., 2017: 4768–77.
