# Supplementary material for: Deep learning to predict rapid progression of Alzheimer’s disease from pooled clinical trials: A retrospective study
Source: PLOS Digit Health. 2024 Apr 10;3(4):e0000479. doi: 10.1371/journal.pdig.0000479 (PMC11006164; doi:10.1371/journal.pdig.0000479)
Supplement: S1 Fig — (A) Baseline variables: mean imputation. Longitudinal variables: 1) last observation carried forward (LOCF); 2) linear imputation; and, 3) multiple imputation by chained equation (MICE). (B) Baseline variables: MICE. Longitudinal variables: 1) LOCF; 2) linear imputation; and, 3) MICE. (C) Baseline variables: 1) mean imputation; and, 2) MICE. Longitudinal variables: MICE. (DOCX) [file pdig.0000479.s002.docx]

**S1 Fig. AUROCs and AUPRCs of RP prediction models of different imputation strategies.**

(A) Baseline variables: mean imputation. Longitudinal variables: 1) last observation carried forward (LOCF); 2) linear imputation; and, 3) multiple imputation by chained equation (MICE). (B) Baseline variables: MICE. Longitudinal variables: 1) LOCF; 2) linear imputation; and, 3) MICE. (C) Baseline variables: 1) mean imputation; and, 2) MICE. Longitudinal variables: MICE.
