# Supplementary material for: Deep learning to predict rapid progression of Alzheimer’s disease from pooled clinical trials: A retrospective study
Source: PLOS Digit Health. 2024 Apr 10;3(4):e0000479. doi: 10.1371/journal.pdig.0000479 (PMC11006164; doi:10.1371/journal.pdig.0000479)

**S2 Fig. GMM fitting on the ADAS-Cog14, ADCS-ADL, CDR-SB, and MMSE to determine the four rapid progressor definitions.**

The red dash lines indicate the various thresholds. The probability densities of the major Gaussian distribution at the thresholds of CDR-SB, ADAS-Cog14, MMSE, and ADCS-ADL are 0.00094, 0.00041, 0.0014, and 0.0023 respectively, which are close to zero, indicating that the thresholds selected can separate samples within the major distribution and those within the tails. The corresponding areas of tails cut by the thresholds are 0.0019, 0.00077, 0.00061, and 0.0022 respectively.


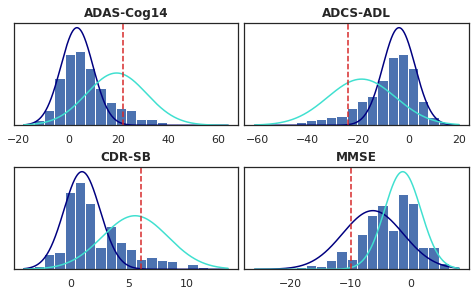

Supplement: S2 Fig — The red dash lines indicate the various thresholds. The probability densities of the major Gaussian distribution at the thresholds of CDR-SB, ADAS-Cog14, MMSE, and ADCS-ADL are 0.00094, 0.00041, 0.0014, and 0.0023, respectively, which are close to zero, indicating that the thresholds selected can separate samples within the major distribution and those within the tails. The corresponding areas of tails cut by the thresholds are 0.0019, 0.00077, 0.00061, and 0.0022, respectively. (DOCX) [file pdig.0000479.s003.docx]
