# Supplementary material for: Deep learning to predict rapid progression of Alzheimer’s disease from pooled clinical trials: A retrospective study
Source: PLOS Digit Health. 2024 Apr 10;3(4):e0000479. doi: 10.1371/journal.pdig.0000479 (PMC11006164; doi:10.1371/journal.pdig.0000479)
Supplement: S4 Fig — ECE: Expected Calibration Error; BS: Brier Score. (DOCX) [file pdig.0000479.s005.docx]

**S4 Fig. Calibration curves for all calibrated models.**

ECE: Expected Calibration Error; BS: Brier Score.
