# Supplementary material for: Deep learning to predict rapid progression of Alzheimer’s disease from pooled clinical trials: A retrospective study
Source: PLOS Digit Health. 2024 Apr 10;3(4):e0000479. doi: 10.1371/journal.pdig.0000479 (PMC11006164; doi:10.1371/journal.pdig.0000479)
Supplement: S1 Table — (DOCX) [file pdig.0000479.s006.docx]

**S1 Table. All variables used in the model training.**

| **Description** | **Variable** | **Modality** |
| --- | --- | --- |
|  |  |  |
| Anticholinergic Medication Use at Baseline | ANTICLNG | Demographics |
| BMI Group | BMIGRP_185_25_30 | Demographics |
| Disease Onset Years | DURDISONSYR | Demographics |
| Years of Formal Education | EDCCNT | Demographics |
| Ethnicity - ﻿Hispanic or Latino | ETHNICLNM | Demographics |
| MMSE Total Score Visit 1 | MMSETSV1 | Demographics |
| MMSE Total Score Visit 2 | MMSETSV2 | Demographics |
| Sex - Female | SEXSNM_F | Demographics |
| Work Status | WORK | Demographics |
| Apolipoprotein E (ApoE) 2/2 | APOEGN_E2/E2 | Demographics |
| Apolipoprotein E (ApoE) 2/3 | APOEGN_E2/E3 | Demographics |
| Apolipoprotein E (ApoE) 2/4 | APOEGN_E2/E4 | Demographics |
| Apolipoprotein E (ApoE) 3/3 | APOEGN_E3/E3 | Demographics |
| Apolipoprotein E (ApoE) 3/4 | APOEGN_E3/E4 | Demographics |
| Apolipoprotein E (ApoE) 4/4 | APOEGN_E4/E4 | Demographics |
| Race - Asian | RACELNM_Asian | Demographics |
| Race - Black or African American | RACELNM_Black or African American | Demographics |
| Race - White | RACELNM_White | Demographics |
| Baseline Acetylcholinesterase Inhibitors (ACHEI) Use | TRTBLGRP_AChEI and Memantine | Demographics |
| Baseline Acetylcholinesterase Inhibitors (ACHEI) Use | TRTBLGRP_AChEI only | Demographics |
| Baseline Memantine Use | TRTBLGRP_Memantine only | Demographics |
| No Baseline Acetylcholinesterase Inhibitors (ACHEI) Use | TRTBLGRP_No SOC Treatment | Demographics |
| Other antiobesity drugs | RX_A08AX | Comorbidities and medications |
| Biguanides | RX_A10BA | Comorbidities and medications |
| Sulfonylureas | RX_A10BB | Comorbidities and medications |
| Dipeptidyl peptidase 4 (dpp-4) inhibitors | RX_A10BH | Comorbidities and medications |
| Vitamin d and analogues | RX_A11CC | Comorbidities and medications |
| Vitamin b-complex, plain | RX_A11EA | Comorbidities and medications |
| Ascorbic acid (vitamin c), plain | RX_A11GA | Comorbidities and medications |
| Calcium, combinations with vitamin d and/or other | RX_A12AX | Comorbidities and medications |
| Iron bivalent, oral preparations | RX_B03AA | Comorbidities and medications |
| Vitamin b12 (cyanocobalamin and analogues) | RX_B03BA | Comorbidities and medications |
| Folic acid and derivatives | RX_B03BB | Comorbidities and medications |
| Alpha-adrenoreceptor antagonists | RX_C02CA | Comorbidities and medications |
| Beta blocking agents, non-selective | RX_C07AA | Comorbidities and medications |
| Beta blocking agents, selective | RX_C07AB | Comorbidities and medications |
| Alpha and beta blocking agents | RX_C07AG | Comorbidities and medications |
| Ace inhibitors, plain | RX_C09AA | Comorbidities and medications |
| Angiotensin ii antagonists, plain | RX_C09CA | Comorbidities and medications |
| Angiotensin ii antagonists and diuretics | RX_C09DA | Comorbidities and medications |
| Hmg coa reductase inhibitors | RX_C10AA | Comorbidities and medications |
| Alpha-adrenoreceptor antagonists | RX_G04CA | Comorbidities and medications |
| Thyroid hormones | RX_H03AA | Comorbidities and medications |
| Acetic acid derivatives and related substances | RX_M01AB | Comorbidities and medications |
| Oxicams | RX_M01AC | Comorbidities and medications |
| Other antiinflammatory and antirheumatic agents, non-steroids | RX_M01AX | Comorbidities and medications |
| Benzodiazepine derivatives | RX_N03AE | Comorbidities and medications |
| Diazepines, oxazepines, thiazepines and oxepines | RX_N05AH | Comorbidities and medications |
| Other antipsychotics | RX_N05AX | Comorbidities and medications |
| Benzodiazepine derivatives | RX_N05BA | Comorbidities and medications |
| Benzodiazepine derivatives | RX_N05CD | Comorbidities and medications |
| Benzodiazepine related drugs | RX_N05CF | Comorbidities and medications |
| Melatonin receptor agonists | RX_N05CH | Comorbidities and medications |
| Non-selective monoamine reuptake inhibitors | RX_N06AA | Comorbidities and medications |
| Selective serotonin reuptake inhibitors | RX_N06AB | Comorbidities and medications |
| Other antidepressants | RX_N06AX | Comorbidities and medications |
| Beta blocking agents | RX_S01ED | Comorbidities and medications |
| Prostaglandin analogues | RX_S01EE | Comorbidities and medications |
| Antiinflammatory preparations, non-steroids for to | RX_M02AA | Comorbidities and medications |
| Coronary artery bypass | DX_10011077 | Comorbidities and medications |
| Depression | DX_10012378 | Comorbidities and medications |
| Transient ischaemic attack | DX_10044390 | Comorbidities and medications |
| Anticholinesterases | RX_N06DA | Comorbidities and medications |
| Other anti-dementia drugs | RX_N06DX | Comorbidities and medications |
| Language | ADAS_ADASS1 | Neurocognitive measures |
| Word Recall Task | ADAS_ADASS10 | Neurocognitive measures |
| Word Recognition Task | ADAS_ADASS11 | Neurocognitive measures |
| Delayed Word Recall | ADAS_ADASS12 | Neurocognitive measures |
| Executive Function (Maze) errors | ADAS_ADASS13A | Neurocognitive measures |
| Executive Function (Maze) time | ADAS_ADASS13B | Neurocognitive measures |
| Number Cancellation: Number of Targets Hit | ADAS_ADASS14A | Neurocognitive measures |
| Number Cancellation: Number of Errors | ADAS_ADASS14B | Neurocognitive measures |
| Number Cancellation: Number of Times Reminded of Task | ADAS_ADASS14C | Neurocognitive measures |
| Comprehension of Spoken Language | ADAS_ADASS2 | Neurocognitive measures |
| Remembering Test Instructions | ADAS_ADASS3 | Neurocognitive measures |
| Word-finding Difficulty | ADAS_ADASS4 | Neurocognitive measures |
| Commands | ADAS_ADASS5 | Neurocognitive measures |
| Naming Objects and Fingers | ADAS_ADASS6 | Neurocognitive measures |
| Constructional Praxis | ADAS_ADASS7 | Neurocognitive measures |
| Ideational Praxis | ADAS_ADASS8 | Neurocognitive measures |
| Orientation | ADAS_ADASS9 | Neurocognitive measures |
| ADL Summary Score of Alone | ADL_ADLALONES | Quality of life |
| ADL Summary Score of Basic | ADL_ADLBASES | Quality of life |
| ADL Summary Score of Current | ADL_ADLCURREVS | Quality of life |
| ADL Summary Score of ADLS6 and ADLS7 | ADL_ADLDRESSS | Quality of life |
| ADL Summary Score of Hobby | ADL_ADLHOBBYS | Quality of life |
| ADL Summary Score of Instrumental | ADL_ADLINSTS | Quality of life |
| ADL Summary Score of Read | ADL_ADLREADS | Quality of life |
| ADL Summary Score of ADLS19 and ADLPAYFLG | ADL_ADLSHOPS | Quality of life |
| ADL Summary Score of ADLS9, ADLS10, and ADL11 | ADL_ADLTELEVSNS | Quality of life |
| Memory | CDR_CDRS86 | Neurocognitive measures |
| Orientation | CDR_CDRS87 | Neurocognitive measures |
| Judgment and Problem Solving | CDR_CDRS88 | Neurocognitive measures |
| Community Affairs | CDR_CDRS89 | Neurocognitive measures |
| Home and Hobbies | CDR_CDRS90 | Neurocognitive measures |
| Personal Care | CDR_CDRS91 | Neurocognitive measures |
| MMSE Attention and Calculation | MMSE_MMSEACS | Neurocognitive measures |
| MMSE Language | MMSE_MMSELNGS | Neurocognitive measures |
| MMSE Orientation | MMSE_MMSEORIS | Neurocognitive measures |
| MMSE Registration | MMSE_MMSEREGS | Neurocognitive measures |
| MMSE Recall | MMSE_MMSERS | Neurocognitive measures |
| NPI Appetite and Eating Disorders Total Score | NPI_NPIAEDTS | Neuropsychiatric symptoms |
| NPI Agitation Total Score | NPI_NPIAGIT8TS | Neuropsychiatric symptoms |
| NPI Apathy/Indifference Total Score | NPI_NPIAITS | Neuropsychiatric symptoms |
| NPI Aberrant Motor Behavior Total Score | NPI_NPIAMBTS | Neuropsychiatric symptoms |
| NPI Anxiety Total Score | NPI_NPIANXTS | Neuropsychiatric symptoms |
| NPI Delusions Total Score | NPI_NPIDELUSTS | Neuropsychiatric symptoms |
| NPI Disinhibition Total Score | NPI_NPIDISINHIBTS | Neuropsychiatric symptoms |
| NPI Depression Total Score | NPI_NPIDPSNTS | Neuropsychiatric symptoms |
| NPI Caregiver Distress Total Score | NPI_NPIDTRTS | Neuropsychiatric symptoms |
| NPI Elation/Euphoria Total Score | NPI_NPIELEUTS | Neuropsychiatric symptoms |
| NPI Frequency Rating Total Score | NPI_NPIFREQTS | Neuropsychiatric symptoms |
| NPI Hallucinations Total Score | NPI_NPIHALUCIN8TS | Neuropsychiatric symptoms |
| NPI Irritability Total Score | NPI_NPIIRRTTS | Neuropsychiatric symptoms |
| NPI Severity Rating Total Score | NPI_NPISEVTS | Neuropsychiatric symptoms |
| NPI Sleep and Nighttime Behavior Disorders Total Score | NPI_NPISNBDTS | Neuropsychiatric symptoms |
| Visual Analog Scale Health State Score | EQ5D_EQ5HASS | Quality of life |
| Mobility | EQ5D_EQ5S1 | Quality of life |
| Self-Care | EQ5D_EQ5S2 | Quality of life |
| Usual Activities (e.g. work, study, housework, family or leisure activities) | EQ5D_EQ5S3 | Quality of life |
| Pain/Discomfort | EQ5D_EQ5S4 | Quality of life |
| Anxiety/Depression | EQ5D_EQ5S5 | Quality of life |
| EQ-5D UK population-based Index Score | EQ5D_EQ5UKIXS | Quality of life |
| EQ-5D US population-based Index Score | EQ5D_EQ5USIXS | Quality of life |
| Physical Health | QLADC_QLADCARES1 | Quality of life |
| Ability to do chores <around their room> | QLADC_QLADCARES10 | Quality of life |
| Ability to do things for fun | QLADC_QLADCARES11 | Quality of life |
| Money | QLADC_QLADCARES12 | Quality of life |
| Life as a whole | QLADC_QLADCARES13 | Quality of life |
| Energy | QLADC_QLADCARES2 | Quality of life |
| Mood | QLADC_QLADCARES3 | Quality of life |
| Living Situation | QLADC_QLADCARES4 | Quality of life |
| Memory | QLADC_QLADCARES5 | Quality of life |
| Family | QLADC_QLADCARES6 | Quality of life |
| Marriage | QLADC_QLADCARES7 | Quality of life |
| Friends | QLADC_QLADCARES8 | Quality of life |
| Self as whole | QLADC_QLADCARES9 | Quality of life |
| Composite Summary SUVR, mean by whole cerebellum | PET_SUVR |  |
| Left entorhinal cortex volume | MRI_ERCV_L | Brain volumetry |
| Right entorhinal cortex volume | MRI_ERCV_R | Brain volumetry |
| Left hippocampus volume | MRI_HV_L | Brain volumetry |
| Right hippocampus volume | MRI_HV_R | Brain volumetry |
| Ventricular volume | MRI_VV | Brain volumetry |
| Whole brain volume | MRI_WBV | Brain volumetry |
| plasma level Aβ_1-42_/Aβ_1-40_ ratio | LB_amyloid42/40 | Plasma level Aβ |
| Body Mass Index | VS_BMI |  |
| BP Diastolic Result | VS_DIA |  |
| Pulse Rate Result | VS_PLS |  |
| BP Systolic Result | VS_SYS |  |
| Temperature Result | VS_TEMP |  |
| Age | AGE |  |
