# Supplementary material for: Deep learning to predict rapid progression of Alzheimer’s disease from pooled clinical trials: A retrospective study
Source: PLOS Digit Health. 2024 Apr 10;3(4):e0000479. doi: 10.1371/journal.pdig.0000479 (PMC11006164; doi:10.1371/journal.pdig.0000479)
Supplement: S2 Table — (DOCX) [file pdig.0000479.s007.docx]

**S2 Table. Mapping between variables and neurocognitive domains**.

| **Domain** | **Test** | **Variable** | **Description** |
| --- | --- | --- | --- |
| Complex Attention | ADAS | ADAS_ADASS14A | Number Cancellation: Number of Targets Hit |
|  |  | ADAS_ADASS14B | Number Cancellation: Number of Errors |
|  |  | ADAS_ADASS14C | Number Cancellation: Number of Times Reminded of Task |
|  |  | ADAS_ADASS3 | Remembering Test Instructions |
|  |  | ADAS_ADASS5 | Commands |
|  | CDR | CDR_CDRS90 | Home and Hobbies |
|  | MMSE | MMSE_MMSEACS | MMSE Attention and Calculation |
| Executive Function | ADAS | ADAS_ADASS13A | Executive Function (Maze) errors |
|  |  | ADAS_ADASS13B | Executive Function (Maze) time |
|  | CDR | CDR_CDRS88 | Judgment and Problem Solving |
|  |  | CDR_CDRS89 | Community Affairs |
|  |  | CDR_CDRS90 | Home and Hobbies |
|  |  | CDR_CDRS91 | Personal Care |
|  | MMSE | MMSE_MMSEACS | MMSE Attention and Calculation |
| Language | ADAS | ADAS_ADASS1 | Language |
|  |  | ADAS_ADASS2 | Comprehension of Spoken Language |
|  |  | ADAS_ADASS4 | Word-finding Difficulty |
|  |  | ADAS_ADASS6 | Naming Objects and Fingers |
|  | MMSE | MMSE_MMSELNGS | MMSE Language |
| Learning and Memory | ADAS | ADAS_ADASS10 | Word Recall Task |
|  |  | ADAS_ADASS11 | Word Recognition Task |
|  |  | ADAS_ADASS12 | Delayed Word Recall |
|  |  | ADAS_ADASS9 | Orientation |
|  | CDR | CDR_CDRS86 | Memory |
|  |  | CDR_CDRS87 | Orientation |
|  |  | CDR_CDRS91 | Personal Care |
|  | MMSE | MMSE_MMSEORIS | MMSE Orientation |
|  |  | MMSE_MMSEREGS | MMSE Registration |
|  |  | MMSE_MMSERS | MMSE Recall |
| Motor Function | ADAS | ADAS_ADASS7 | Constructional Praxis |
|  |  | ADAS_ADASS8 | Ideational Praxis |
| Social Cognition | CDR | CDR_CDRS89 | Community Affairs |
