# Supplementary material for: Deep learning to predict rapid progression of Alzheimer’s disease from pooled clinical trials: A retrospective study
Source: PLOS Digit Health. 2024 Apr 10;3(4):e0000479. doi: 10.1371/journal.pdig.0000479 (PMC11006164; doi:10.1371/journal.pdig.0000479)
Supplement: S3 Table — (DOCX) [file pdig.0000479.s008.docx]

**S3 Table. Number and percentage of rapid progressors separated by the four rapid progressor definitions.**

| **Definitions** | **EXPEDITION**  **(n=368)** | **EXPEDITION 2**  **(n=391)** | **EXPEDITION 3**  **(n=844)** | **Pooled**  **(n=1603)** |
| --- | --- | --- | --- | --- |
| ADAS-Cog14 > 22 | 36 (9.8%) | 45 (11.5%) | 66 (7.8%) | 147 (9.2%) |
| ADCS-ADL < -24 | 35 (9.5%) | 53 (13.6%) | 64 (7.6%) | 152 (9.5%) |
| CDR-SB > 6 | 35 (9.5%) | 44 (11.3%) | 71 (8.4%) | 150 (9.4%) |
| MMSE < -10 | 32 (8.7%) | 38 (9.7%) | 75 (8.9%) | 145 (9.0%) |
