# Supplementary material for: Deep learning to predict rapid progression of Alzheimer’s disease from pooled clinical trials: A retrospective study
Source: PLOS Digit Health. 2024 Apr 10;3(4):e0000479. doi: 10.1371/journal.pdig.0000479 (PMC11006164; doi:10.1371/journal.pdig.0000479)
Supplement: S4 Table — (DOCX) [file pdig.0000479.s009.docx]

**S4 Table.** **Results of AUROC and AUPRC on the internal and external test datasets.**

|  | | **ADAS-Cog14** | | **ADCS-ADL** | | **CDR-SB** | | **MMSE** | |
| --- | --- | --- | --- | --- | --- | --- | --- | --- | --- |
|  |  | **AUROC** | **AUPRC** | **AUROC** | **AUPRC** | **AUROC** | **AUPRC** | **AUROC** | **AUPRC** |
| **BL** | **In** | 0.78 (0.77–0.79) | 0.28 (0.26–0.29) | 0.74 (0.72–0.76) | 0.25 (0.24–0.27) | 0.75 (0.73–0.76) | 0.26 (0.24–0.28) | 0.79 (0.78–0.81) | 0.40 (0.38­–0.42) |
|  | **Ex** | 0.72 (0.68–0.76) | 0.28 (0.24–0.31) | 0.68 (0.64–0.71) | 0.21 (0.19–0.24) | 0.68 (0.67–0.69) | 0.19 (0.18–0.19) | 0.73 (0.70–0.75) | 0.31 (0.27–0.35) |
| **Wk 4** | **In** | 0.73 (0.72–0.74) | 0.25 (0.23–0.27) | 0.68 (0.62–0.74) | 0.21 (0.18–0.24) | 0.71 (0.69–0.73) | 0.21 (0.20–0.23) | 0.77 (0.75–0.78) | 0.35 (0.33–0.38) |
|  | **Ex** | 0.73 (0.71–0.74) | 0.27 (0.26–0.29) | 0.68 (0.65–0.70) | 0.21 (0.19–0.23) | 0.68 (0.65–0.70) | 0.18 (0.16–0.21) | 0.74 (0.73–0.75) | 0.34 (0.33–0.36) |
| **Wk 12** | **In** | 0.77 (0.75–0.78) | 0.33 (0.32–0.35) | 0.73 (0.72–0.74) | 0.25 (0.23–0.27) | 0.71 (0.70–0.72) | 0.25 (0.23–0.26) | 0.78 (0.76–0.79) | 0.36 (0.35–0.38) |
|  | **Ex** | 0.75 (0.73–0.77) | 0.31 (0.28–0.34) | 0.69 (0.67–0.71) | 0.20 (0.19–0.22) | 0.65 (0.60–0.70) | 0.18 (0.15–0.20) | 0.75 (0.74–0.76) | 0.34 (0.32–0.35) |
| **Wk 28** | **In** | 0.82 (0.82–0.83) | 0.44 (0.41–0.46) | 0.80 (0.78–0.83) | 0.38 (0.35–0.41) | 0.81 (0.79–0.82) | 0.34 (0.32–0.36) | 0.80 (0.79–0.82) | 0.46 (0.44–0.49) |
|  | **Ex** | 0.83 (0.82–0.84) | 0.45 (0.43–0.48) | 0.77 (0.75–0.78) | 0.29 (0.27–0.31) | 0.76 (0.75–0.77) | 0.27 (0.25–0.29) | 0.75 (0.70–0.81) | 0.35 (0.30–0.41) |
| **Wk 40** | **In** | 0.84 (0.82–0.86) | 0.54 (0.52–0.55) | 0.85 (0.83–0.87) | 0.47 (0.45–0.49) | 0.79 (0.76–0.82) | 0.36 (0.33–0.39) | 0.82 (0.80–0.84) | 0.46 (0.44–0.49) |
|  | **Ex** | 0.85 (0.84–0.86) | 0.54 (0.51–0.57) | 0.77 (0.72–0.81) | 0.33 (0.28–0.38) | 0.78 (0.76–0.79) | 0.31 (0.28–0.33) | 0.81 (0.80–0.82) | 0.41 (0.40–0.42) |
| **Wk 52** | **In** | 0.88 (0.86–0.91) | 0.62 (0.59–0.65) | 0.88 (0.87–0.90) | 0.55 (0.53–0.58) | 0.86 (0.84–0.88) | 0.49 (0.47–0.51) | 0.83 (0.82–0.84) | 0.55 (0.52–0.57) |
|  | **Ex** | 0.89 (0.88–0.90) | 0.61 (0.60–0.62) | 0.83 (0.81–0.84) | 0.44 (0.42–0.46) | 0.83 (0.81–0.84) | 0.40 (0.38–0.41) | 0.84 (0.82–0.85) | 0.48 (0.46–0.50) |
| **Wk 64** | **In** | 0.90 (0.88–0.91) | 0.68 (0.65–0.71) | 0.91 (0.89–0.92) | 0.67 (0.64–0.69) | 0.86 (0.85–0.88) | 0.51 (0.49–0.54) | 0.84 (0.83–0.86) | 0.58 (0.55–0.61) |
|  | **Ex** | 0.91 (0.89–0.92) | 0.66 (0.63–0.69) | 0.85 (0.84–0.87) | 0.51 (0.48–0.54) | 0.84 (0.83–0.85) | 0.44 (0.42–0.45) | 0.86 (0.85–0.87) | 0.52 (0.50–0.54) |

BL: Baseline; In: Internal validation; Ex: External validation; Wk: Week.
