# Supplementary material for: Deep learning to predict rapid progression of Alzheimer’s disease from pooled clinical trials: A retrospective study
Source: PLOS Digit Health. 2024 Apr 10;3(4):e0000479. doi: 10.1371/journal.pdig.0000479 (PMC11006164; doi:10.1371/journal.pdig.0000479)
Supplement: S5 Table — (DOCX) [file pdig.0000479.s010.docx]

**S5 Table. Frameworks and libraries used for building, training, and evaluating the models.**

| GPU | NVIDIA V100 |
| --- | --- |
| Container image | NVIDIA PyTorch container image 22.12 |
| CUDA | 11.8.0 |
| Python | 3.8.10 |
| PyTorch | 1.14.0a0+410ce96 |
| Scikit-learn | 1.3.2 |
| Numpy | 1.22.2 |
| Pandas | 1.5.2 |
| Matplotlib | 3.7.3 |
| Captum | 0.6.0 |
