# Supplementary material for: Deep learning to predict rapid progression of Alzheimer’s disease from pooled clinical trials: A retrospective study
Source: PLOS Digit Health. 2024 Apr 10;3(4):e0000479. doi: 10.1371/journal.pdig.0000479 (PMC11006164; doi:10.1371/journal.pdig.0000479)
Supplement: S6 Table — (DOCX) [file pdig.0000479.s011.docx]

**S6 Table. TRIPOD checklist of this study.**

| Section/topic | Item | Development or validation? | Checklist item | Section/Paragraph |
| --- | --- | --- | --- | --- |
| Title and abstract |  |  |  |  |
| Title | 1 | D;V | Identify the study as developing and/or validating a multivariable prediction model, the target population, and the outcome to be predicted. | Title |
| Abstract | 2 | D;V | Summarizes objectives, study design, setting, participants, sample size, predictors, outcome, statistical analysis, results, and conclusions. | “Abstract” and “Summary” sections |
| Introduction |  |  |  |  |
| Background and objectives | 3a | D;V | Explain the medical context (including whether diagnostic or prognostic) and rationale for developing or validating the multivariable prediction model, including references to existing models. | Paragraphs 1–3 in the “Introduction” section |
|  | 3b | D;V | Specify the objectives, including whether the study describes the development or validation of the model or both. | Paragraph 4 in the “Introduction” section |
| Methods |  |  |  |  |
| Source of data | 4a | D;V | Describe the study design or data source (e.g., randomized trial, cohort, or registry data), separately for the development and validation data sets, if applicable. | “Data source” subsection in the “Materials and Methods” section |
|  | 4b | D;V | Specify the key study dates, including start of accrual; end of accrual; and, if applicable, end of follow-up. | “Data source” subsection in the “Materials and Methods” section |
| Participants | 5a | D;V | Specify key elements of the study setting (e.g., primary care, secondary care, general population) including number and location of centers. | N/A |
|  | 5b | D;V | Describe eligibility criteria for participants. | “Data source” subsection in the “Materials and Methods” section |
|  | 5c | D;V | Give details of treatments received, if relevant. | N/A |
| Outcome | 6a | D;V | Clearly define the outcome predicted by the prediction model, including how and when assessed. | “Definitions of a rapid progressor” subsection in the “Materials and Methods” section |
|  | 6b | D;V | Report any actions to blind assessment of the outcome to be predicted. | N/A |
| Predictors | 7a | D;V | Clearly define all predictors used in developing the multivariable prediction model, including how and when they were measured. | “Variables” subsection in the “Materials and Methods” section |
|  | 7b | D;V | Report any actions to blind assessment of predictors for the outcome and other predictors. | N/A |
| Sample size | 8 | D;V | Explain how the study size arrived. | “Data processing” subsection in the “Materials and Methods” section |
| Missing data | 9 | D;V | Describe how missing data were handled (e.g., complete-case analysis, single imputation, multiple imputation) with details of any imputation method. | “Data processing” subsection in the “Materials and Methods” section |
| Statistical analysis methods | 10a | D | Describe how predictors were handled in the analyses. | “Data processing” subsection in the “Materials and Methods” section |
|  | 10b | D | Specify the type of model, all model-building procedures (including any predictor selection), and method for internal validation. | “Prediction model”, and “Evaluation” subsections in the “Materials and Methods” section |
|  | 10c | V | For validation, describe how the predictions were calculated. | “Evaluation” subsection in the “Materials and Methods” section |
|  | 10d | D;V | Specify all measures used to assess model performance and, if relevant, to compare multiple models. | “Evaluation” subsection in the “Materials and Methods” section |
|  | 10e | V | Describe any model updating (e.g., recalibration) arising from the validation if done. | N/A |
| Risk groups | 11 | D;V | Provide details on how risk groups were created if done. | N/A |
| Development vs. validation | 12 | V | For validation, identify any differences from the development data in setting, eligibility criteria, outcome, and predictors. | “Evaluation” subsection in the “Materials and Methods” section |
| Results |  |  |  |  |
| Participants | 13a | D;V | Describe the flow of participants through the study, including the number of participants with and without the outcome and, if applicable, a summary of the follow-up time. A diagram may be helpful. | N/A |
|  | 13b | D;V | Describe the characteristics of the participants (basic demographics, clinical features, available predictors), including the number of participants with missing data for predictors and outcome. | “Rapid progressors” subsection in the “Results” section |
|  | 13c | V | For validation, show a comparison with the development data of the distribution of important variables (demographics, predictors and outcome). | “Data” and “Rapid progressors” subsections in the “Results” section |
| Model development | 14a | D | Specify the number of participants and outcome events in each analysis. | “Data” and “Rapid progressors” subsections in the “Results” section |
|  | 14b | D | If done, report the unadjusted association between each candidate predictor and outcome. | N/A |
| Model specification | 15a | D | Present the full prediction model to allow predictions for individuals (i.e., all regression coefficients, and model intercept or baseline survival at a given time point). | “Observation periods”, “Prediction model”, “Evaluation”, and “Interpretation” subsections in the “Materials and Methods” section |
|  | 15b | D | Explain how to use the prediction model. | “Prediction model” subsection in the “Materials and Methods” section |
| Model performance | 16 | D;V | Report performance measures (with CIs) for the prediction model. | “Rapid progressor prediction” subsection in the “Results” section. |
| Model updating | 17 | V | If done, report the results from any model updating (i.e., model specification, model performance). | N/A |
| Discussion |  |  |  |  |
| Limitations | 18 | D;V | Discuss any limitations of the study (such as nonrepresentative sample, few events per predictor, missing data). | Paragraphs 9–11 in the “Discussion” section |
| Interpretation | 19a | V | For validation, discuss the results with reference to performance in the development data, and any other validation data. | Paragraphs 2–6 in the “Discussion” section |
|  | 19b | D;V | Give an overall interpretation of the results, considering objectives, limitations, results from similar studies, and other relevant evidence. | Paragraph 1 in the “Discussion” section |
| Implications | 20 | D,V | Discuss the potential clinical use of the model and implications for future research. | Paragraphs 7–8 in the “Discussion” section |
| Other information | | |  |  |
| Supplementary information | 21 | D;V | Provide information about the availability of supplementary resources, such as study protocol, Web calculator, and data sets. | “Supporting Information” section |
| Funding | 22 | D;V | Give the source of funding and the role of the funders for the present study. | “Funding” section |

*Items relevant only to the development of a prediction model are denoted by D, items relating solely to validation of a prediction model are denoted by V, and items relating to both are denoted D;V.
