# Supplementary material for: Deep learning to predict rapid progression of Alzheimer’s disease from pooled clinical trials: A retrospective study
Source: PLOS Digit Health. 2024 Apr 10;3(4):e0000479. doi: 10.1371/journal.pdig.0000479 (PMC11006164; doi:10.1371/journal.pdig.0000479)
Supplement: Attachment — Submitted filename: Peer_Review_PLOS_Digital_Health____Machine_learning_to_predict_rapid_progression_of_Alzheimer_s_disease_from_pooled_clinical_trials.pdf [file pdig.0000479.s012.pdf]

Peer Review

Machine learning to predict rapid progression of Alzheimer's disease from pooled clinical trials

Simon Provost, sgp28@kent.ac.uk
Ph.D. Student at the University of Kent, United-Kingdom

1 Research Overview and Initial Impressions

In this retrospective study, the authors take advantage of machine learning, exploring its capabilities to predict the rapid progression of Alzheimer's disease (AD) using pooled data from clinical trials. The premise of the paper is based on the known variation in progression rates among AD patients, highlighting the significance of identifying rapid progressors (RPs) for the effective design of clinical trials and the customisation of treatments. My interpretation of this is that such a disease's challenge suggests that RPs might be perceived as "biassed" or "outliers" in clinical trials focused on ADs. Consequently, integrating data from RPs with those from non-RPs could compromise the reliability of the trial outcomes, leading one to mistakenly attribute findings exclusively to AD progression when, in fact, RPs exhibit distinct disease behaviour, yet the same disease. Therefore, the identification of RPs and perhaps removing or categorising them with the distinct binary feature "is RPs", could prove worthiness for further clinical trials in this direction. Lastly, such prediction-accurate-based models could pave the way for targeted pre-treatments or other clinical interventions specifically addressing the unique challenges posed by RPs.

My first initial impression is in the data acquisition's stage: The researchers combined de-identified placebo-arm data from three well-known randomised controlled trials (RCTs) — EXPEDITION, EXPEDITION 2, and EXPEDITION 3 — which, after processing, included 1,603 participants. This data, generously provided by Eli Lilly and Company, extends 80 weeks and contains extensive longitudinal information on neurocognitive health, brain volumes, and amyloid-beta levels in patients with mild-to-moderate AD. Moreover, the definition of RPs was based on four neurocognitive and functional health measures. Therefore, as a result and as a reader, I am extremely grateful for the "quantity" of data utilised throughout the study. Even in a healthcare setting, the great volume of data and its temporal depth are commendable, as evidenced not only by the number of participants but also their longitudinal characteristics. It instills confidence in the reader and bolsters the paper's credibility, compelling one to investigate its contents further. As a result, evident methodological rigour is present.

While the stage of data acquisition has been conducted with methodological rigour and precision, **my analytical approach impression is as follow**: The authors made use of sophisticated recurrent neural networks with attention modules, demonstrating their commitment to producing accurate and reliable predictions while taking into consideration the temporal dependency the data provides to the challenge they are trying to tackle. The attention modules merely permit a greater emphasis on more recent data than older data, implying that recent data are more relevant in this medical context (longitudinal data and Alzheimer). As a result, the models, particularly those trained with 28 weeks of observation, received high validation scores, which shows great promise. In addition, the study did not solely focus on prognosis; it also identified the main factors that played a significant role in these predictions, including regional brain volumetry and various neurocognitive health metrics. Consequently, the distinction between predicted RP trajectories and non-RP trajectories provides, from a reader's standpoint, a nuanced comprehension that may be necessary for future research and clinical applications in the same medical challenge direction.

The study's conclusion highlights the effectiveness of incorporating clinical data with advanced machine learning techniques. It also acknowledges the potential limitations of the research, such as the necessity to assess the ongoing suitability of this machine learning method for addressing the challenges of the disease using non-RCT-based data. E.g., this evaluation would offer a more comprehensive understanding of utilising machine learning in addressing the aforementioned medical challenges as well as determining the appropriate data type to rely on. Overall, I enjoyed the paper, first because it was straightforward and covered a real-world use case challenge, and second, on a more technical level, I appreciated that the stage of data acquisition was methodologically sound and that the analytical approach showed promise while paving the way for further medical exploration using machine learning in AD and RPs. I finally believe that the paper should be accepted; however, I request that the authors consider the following minor suggestions:

2 Minor Considerations and Proposed Recommendations

The section that follows will discuss three fundamental aspects of recommendations and considerations. First, we will provide a few suggestions that we would expect to enhance the reader's understanding of mostly the choices you made during your study, most likely to be explained in the appendix, though this is not etched in stone; see below for more. Second, we believe that your future work's section may benefit from additional information, but we would like to note that this is entirely debatable. Finally, we would like to call attention to reproducibility, which was not extensively discussed in the paper.

2.1 Enhancing the Appendix for Clarity

Clarifying the Role of the Attention Mechanism

The analytical method utilised in the paper was a recurrent neural network architecture with an additional attention mechanism module. This attention mechanism, as mentioned, is a great standard to use in longitudinal data classification. As you mentioned, it can focus on temporal dependency by, for instance, favouring the most recent time steps information over the older ones, assuming that the patient's status of the most recent time steps is most likely closer than that of the older time steps.

As a machine learning applied medicine individual, I do comprehend these concepts; however, a portion of the journal's readers are likely to have a strong medical background and may be unfamiliar with neural networks and attention mechanisms. While neural network literature and materials are readily available, I believe that attention mechanisms in the context of longitudinal data are at the very least more specialised. Therefore, I would strongly appreciate it if you considered a solution in this direction, if at all feasible. E.g., An appendix enhancement could include a brief subsection describing how attention mechanisms function and how they could be utilised in networks to prioritise more recent longitudinal data over older data. Another option could be a straightforward citation to a paper or survey or something similar that explains it well for any reader who may be interested in applying it to their medical-based work; however, I believe this should be directed towards the attention mechanism for longitudinal data. I would strongly recommend assisting the readers in comprehending the purpose of the attention mechanism in this longitudinal's context, utilising one of the aforementioned proposed solutions or one you deem to be of greater value.

Motive Behind Metric Selection

The metrics of interest in the paper are AUROC and AUPRC. Both are highly utilised analytical metrics in academia and industry. In a medical context, however, when data are imbalanced, AUPRC tends to be more valued than AUROC, despite the fact that there are other motives for this as well.

In the meantime, I would encourage the authors to elaborate on their motives for selecting these two specific metrics. While it is acknowledged that these metrics are frequently used in medical contexts, it is unclear whether the data under consideration are imbalanced or if

other cogent arguments favour their selection. Therefore, to avoid any potential ambiguity, I suggest that the authors either elaborate on this in the main body of the manuscript or in an appendix.

Justification for Evaluation Methodology

The chosen evaluation method, which could be characterised as a hold-out external set validation method, is extremely advantageous in certain circumstances. However, it is simple to engage in the discussion, as a reader, why K-Fold Cross Validation has not been employed instead. While statistically, these evaluation methodologies have advantages and disadvantages, I believe there is a reason you chose the holdout-based validation method over others.

For instance, a straightforward reason could be that this method is more computationally and timely efficient, whereas you may not have had both resources to execute K-fold cross-validation during your study. Another reason I can think of is that standard K-fold cross-validation could result in longitudinal data leakage, which is not necessarily representative of the real-world scenario. However, this is contingent upon the data structure of your longitudinal characteristics, to which I did not have access. Due to the longitudinal characteristics of each patient that manifest across the row direction of a dataset, K-fold cross validation can inadvertently introduce data leakage by splitting information from the same patients, making the method less reliable for real-world scenarios. Consequently, the hold-out external set serves as an excellent alternative for evaluation, as it circumvents the risk of this longitudinal data leakage.

As a result, as a reader, it was unclear why this evaluation method was chosen and for what specific reasons. Hence, it would be greatly appreciated if the manuscript or appendix provided the actual explanation. Again, we believe that you may have additional, potentially superior solutions, so please feel free to act accordingly.

2.2 Future Work Enhancement

Considerations for Missing Value Imputations

While preprocessing techniques have been investigated, carrying forward the last observation remains a frequently used data preprocessing technique for missing values. However, there are many more statistical missing value imputation methods, as well as supervised machine learning methods and data-driven methods for missing value imputation, specifically applicable to longitudinal datasets. While, furthermore, the labour-intensive nature of the exploration is the best for a specific scenario, focused-missing value imputation systems utilising Bayesian optimisation or even a brute-force or random search for finding the best setting for your use case are also available in the literature.

Therefore, while the study shows great promise, the data preprocessing stage, such as missing value imputation, feature selection, etc., is very basic and could benefit from much more involvement. I would appreciate the authors to consider noting out to the reader in the future work section, for instance, that the missing value imputation technique employed is functional, but it is still uncertain if it is the most appropriate, and that future work could benefit from further exploration. By further exploration, here are two examples: one of which

is a data-driven approach that selects (i.e., sort of in a brute force way) the most efficient missing value imputation technique for a longitudinal dataset, and the other is a Bayesian optimisation's system focusing on missing value imputation in general, not necessarily for longitudinal data only, for which I believe the search space could be tailored to include only methods applicable to longitudinal datasets. In the meantime, the first system is less complex in terms of its use of carried-forward and backward-based missing value imputations and supervised-based missing value imputation techniques, and it has been evaluated in a longitudinal classification setting. The second system is more complex because it uses Bayesian optimisation to seek out the most efficient candidate setting under a time constraint [1, 2].

Comparing Adapted Supervised Machine Learning Algorithms *and* Neural Networks

While neural networks (NNs) and the attention mechanism offer a very promising approach to coping with longitudinal data classification, there are still significant challenges to be addressed. Adapted supervised machine learning algorithms appear to have emerged in the literature more recently [3, 4]. The overarching concept is to modify a standard machine learning algorithm, such as a decision tree, random forest, XGBoost, etc., to specifically address the temporality challenge that longitudinal data presents.

As a result, while the study shows great promise, it would be advantageous to note that further exploration between NNs and adapted machine learning algorithms for longitudinal classification could be conducted with the goal of determining which of the two types of models is realistically more efficient for this medical use case, and even further, in a multi-objective analysis, which of the two models outputs the best trade-off between, e.g., accuracy and interpretability. Consequently, I would appreciate it if the authors would consider pointing out to the reader, for instance, in the section on future work, that the investigation of adapted machine learning algorithms for longitudinal classification could be a worthwhile research direction, given the many interpretability and medical context-related benefits that such models can also provide.

3 Promoting Research Reproducibility and Research Open Source Community

While the study demonstrates promise, we would like to emphasise the importance of accelerating research in this direction via reproducibility. Perhaps you are restricted by your funding or the data provider, but code reproducibility is a very promising first step, even though the data needs licencing and an external request by the interested party.

Consequently, I would like the author to consider adding either to the appendix or to the subsections on data availability, contributors, etc., an additional section pertaining to offering the reader a potential open-source hosted code of your architecture and any scripts of your experiments, allowing any interested reader to begin with concrete as opposed to starting from scratch. If you are not permitted or do not have a hosted-based service with your code, such as GitHub, GitLab, etc., for your research, I would kindly understand that it could take

too long to produce such a thing and that, at this stage, this is not a significant point to consider. Despite this, the research community greatly values codes and how to reproduce papers' experiments, such as with scripts; therefore, we highly recommend kindly considering this in the future.

References

[1] C. Ribeiro and A. A. Freitas, "A data-driven missing value imputation approach for longitudinal datasets," *Artificial Intelligence Review*, pp. 1–31, 2021.

[2] D. Jarrett, B. C. Cebere, T. Liu, A. Curth, and M. van der Schaar, "Hyperimpute: Generalized iterative imputation with automatic model selection," in *International Conference on Machine Learning*. PMLR, 2022, pp. 9916–9937.

[3] S. Ovchinnik, F. Otero, and A. A. Freitas, "Nested trees for longitudinal classification," in *Proceedings of the 37th ACM/SIGAPP Symposium on Applied Computing*, 2022, pp. 441–444.

[4] C. Ribeiro and A. Freitas, "A new random forest method for longitudinal data classification using a lexicographic bi-objective approach," in *2020 IEEE Symposium Series on Computational Intelligence (SSCI)*. IEEE, 2020, pp. 806–813.
