# Supplementary material for: Deep learning to predict rapid progression of Alzheimer’s disease from pooled clinical trials: A retrospective study
Source: PLOS Digit Health. 2024 Apr 10;3(4):e0000479. doi: 10.1371/journal.pdig.0000479 (PMC11006164; doi:10.1371/journal.pdig.0000479)
Supplement: Attachment — Submitted filename: Response to Reviewers Final.pdf [file pdig.0000479.s013.pdf]

Dear Reviewers,

We would like to express our heartfelt gratitude for your review and feedback on our manuscript. Below, you will find how we have addressed your feedback, making substantial changes to the manuscript.

We made the following major changes to the manuscript and supporting information:

1. According to Review #3 Comment 4 and Review #4 Comment 3, we reran the whole pipeline using 10-fold cross validation and updated the model accuracy results in the “Abstract” section, “Results” section, and the figures (Fig 2, Fig 4, Fig 5).
2. According to Review #3 Comment 7 and Review #4 Comment 4, we compared several data imputation strategies and reported the methods and results in supporting information S1 Appendix and S2 Fig. We updated our models from using last observation carried forward (LOCF) to multiple imputation by chained equation (MICE) for longitudinal data imputation.
3. According to Review #2 Comment 6, we added a new figure (Fig 3) to show the confusion matrices and F1 scores of our models.
4. According to Review #2 Comment 6, Review #3 Comment 5, and Review #4 Comment 2, we rewrote the “Evaluation” subsection in the “Results” section reflecting the new pipeline for evaluation, including the cross-validation, confusion matrices, F1 scores, and more details of the selected metrics.
5. According to Review #2 Comment 1 and Review #4 Comment 6, we added the GitHub repo with our code for building, training, and evaluating the models. We also reported the specific environment and libraries in the supporting information S5 Table.
6. According to Review #4 Comment 1, we added the description and visualization of attention heat maps in the supporting information S1 Appendix and S3 Fig.
7. Aside from the comments from the reviewers, we updated the Fig 5 (old Fig 4) by reporting predicted positives and predicted negatives instead of true positives and true negatives to show the stratification by our models more accurately. We also included ground-truth positives and negatives for reference. We modified the “Interpretation” subsection of “Materials and methods” section and S1 Appendix accordingly.

Below is the point-by-point response to all comments of the four reviewers.

I. Response to Reviewer #1

- ***(1) The abstract is unstructured it needs re-synthesis and should be structured. (2) The 'Author Summary' should be replaced by 'Summary'. (3) The introduction and discussion needs to be more explanatory.***

  Thank you for raising these points.

  (1) The PLOS Digital Health submission guideline states: “While the Abstract is conceptually divided into three sections (Background, Methodology/Principal Findings, and Conclusions/Significance), do not apply these distinct headings to the Abstract within the article file.” (https://journals.plos.org/digitalhealth/s/submission-guidelines#loc-abstract) Based on this guideline, our revised abstract has the following structure:

1) (Background) “The rate of progression of Alzheimer’s disease (AD) differs dramatically between patients. Identifying the most is critical because when their numbers differ between treated and control groups, it distorts the outcome making it impossible to tell whether the treatment was beneficial. Much recent effort, then, has gone into identifying RPs.”

2) (Methodology) “We pooled de-identified placebo-arm data of three randomized controlled trials (RCTs), EXPEDITION, EXPEDITION 2, and EXPEDITION 3, provided by Eli Lilly and Company. After processing, the data included 1603 mild-to-moderate AD patients with 80 weeks of longitudinal observations on neurocognitive health, brain volumes, and amyloid-beta (Aβ) levels. RPs were defined by changes in four neurocognitive/functional health measures. We built models of recurrent neural networks with attention mechanisms to predict RPs by week 80 based on varying observation periods from baseline (e.g., 12, 28 weeks). Feature importance scores for RP prediction were computed and temporal feature trajectories were compared between RPs and non-RPs. Our evaluation and analysis focused on models trained with 28 weeks of observation.”

3) (Principal findings) “The models achieved robust internal validation area under the receiver operating characteristic (AUROCs) ranging from 0.80 (95% CI 0.79–0.82) to 0.82 (0.81–0.83), and the area under the precision-recall curve (AUPRCs) from 0.34 (0.32–0.36) to 0.46 (0.44–0.49). External validation AUROCs ranged from 0.75 (0.70–0.81) to 0.83 (0.82–0.84) and AUPRCs from 0.27 (0.25–0.29) to 0.45 (0.43–0.48). Aβ plasma levels, regional brain volumetry, and neurocognitive health emerged as important factors for the model prediction. In addition, the trajectories were stratified between predicted RPs and non-RPs based on factors such as ventricular volumes and neurocognitive domains.”

4) (Conclusions/Significance): “Our findings will greatly aid clinical trialists designing tests of new medications, representing a key step towards identifying effective new AD therapies.”

(2) We have replaced the “Author Summary” with “Summary”.

(3) We have edited the introduction and discussion to be more explanatory.

Thank you again for your time and comments on our manuscript.

II. Response to Reviewer #2

1. ***Model/Code transparency: To ensure robustness it is imperative that the exact framework or libraries [that] were used for building, training and evaluating the models is clearly mentioned in the manuscript.***

   Thank you for this comment. To ensure the robustness and transparency of our work, we published the code for building, training and evaluating the models in GitHub (https://github.com/xiaotian0328/AD-Rapid-Progression-Prediction). Specifically, we built a docker container using Nvidia PyTorch container image version 22.12. The main libraries and frameworks we used were Python 3.8.10, CUDA 11.8.0, PyTorch 1.14.0a0+410ce96, Scikit-learn 1.3.2, NumPy 1.22.2, Pandas 1.5.2, Matplotlib 3.7.3, and Captum 0.6.0.

Now, the “Data Availability” section reads:

> Data access restrictions apply. Interested parties may contact Eli Lilly and Company for dataset licensing (https://www.lilly.com/clinical-research/clinical-trials) and request the data following the step-by-step process on the Vivli Platform (https://vivli.org/ourmember/lilly/). Code for building, training, and evaluating the models is available on the GitHub repo (https://github.com/xiaotian0328/AD-Rapid-Progression-Prediction). Specific frameworks and libraries we used are reported in S5 Table.

We added this sentence in the “Overview” section in “Materials and Methods” to guide the readers to this:

> The code for building, training, and evaluating the models can be found on GitHub (https://github.com/xiaotian0328/AD-Rapid-Progression-Prediction).

We also added Table S5 in supporting information reporting the frameworks and libraries for model building, training, and evaluation.

S5 Table. Frameworks and libraries used for building, training, and evaluating the models.

GPU	NVIDIA V100
Container image	NVIDIA PyTorch container image 22.12
CUDA	11.8.0
Python	3.8.10
PyTorch	1.14.0a0+410ce96
Scikit-learn	1.3.2
NumPy	1.22.2
Pandas	1.5.2
Matplotlib	3.7.3
Captum	0.6.0

2. ***Data: Although the data may be proprietary and available on request, a smaller sample de-identified dataset must be made available so that researchers can verify and build upon the current findings. Typically, "available on reasonable request" ends up being most requests being denied.***

   We certainly appreciate the reviewer's comment that the dataset should be made available for verification. Unfortunately, we do not own the dataset and, instead, license it from Eli Lilly and Company. However, just as we applied to Lilly to get the data set, and it was easy to obtain, we would expect that interested researchers could do the same. We clarified this in the manuscript by stating in the “Data Availability” section that “investigators may contact Eli Lilly and Company for dataset licensing (https://www.lilly.com/clinical-research/clinical-trials) and request the data following the step-by-step process on the Vivli Platform (https://vivli.org/ourmember/lilly/).”

3. ***Class imbalance: The proportions of RPs (1) and Not RPs (0) is not clearly mentioned which can help figuring out whether the data is balanced or unbalanced. From Figure 2 it can be seen that AUROC value is quite high whereas AUPRC is low which can be due to unbalanced data. If the data is indeed unbalanced, appropriate measures should be taken to address this.***

   Thank you for raising this point. We agree that it would be beneficial to state the separation of RP and Non-RPs. We have responded by reporting the overall proportions of RPs of the four definitions in the "Rapid progression" subsection of "Results" section, as well as the detailed proportions of RPs in three RCTs in the supporting information (S1 Table).

   We have added a clarifying sentence in the "Rapid progressors" subsection in the "Results" section to include the total amount of RPs versus non-RPs in our sample:

   > After careful consideration of all relevant data per participant, out of 1603 patients, 305 were classified as rapid progressors by at least one of the four definitions.

   To alleviate the imbalance in our dataset, we set the positive weight in PyTorch binary cross entropy loss with logits function to 9 since the rate of negative (non-RP) to positive (RP) samples is approximately 9 based on our definition of RP (10% cut-off results in ~10% RP and ~90% non-RP). This strategy makes the loss act as if there were 9 times more positive samples.

   We clarified this in the supporting information ("Rapid progression prediction model" section in S1 Appendix) by stating the following:

   > The binary cross entropy loss between the output logit $\hat{y}$ and the ground-truth label y is calculated by $l(\hat{y}, y) = -[p \cdot y \cdot \log \sigma(\mathrm{y}) + (1 - y) \cdot \log(1 - \sigma(\hat{y}))]$, where p is the positive weights and $\sigma(\cdot)$ is the sigmoid function. In our setting, $p = 9$ since the ratio between positive and negative samples is approximately 9, which will make the loss act as if there are 9 times more positive samples.

4. ***Study design: The intervals between observation periods are irregular ranging from 4 weeks to 16 weeks. For predicting RPs, the data till 28th week were used but it might be possible to get the same accuracy with 16 weeks or 20 weeks data, this cannot be checked due to the presence of a huge gap between previous observation periods i.e. week 12 and week 28. If this is an issue with data collection this should be clearly mentioned.***

   We greatly appreciate the insightful feedback provided, and we have carefully reviewed and considered your comments. We agree that it is possible that sooner time points would reveal RPs sufficiently. But, as you intuited, the challenge with this is that data was not collected at the 16- and 20-week timepoints. The observation times in the EXPEDITION trials was weeks 0, 4, 12, 28, 40, 52, 64, and 80. These time points were presumably chosen with clinical relevance. The longer and later intervals probably chosen because they are reflective of the expected gradual progression of AD. Our analysis particularly emphasizes the 28-week observation period for our models, as it represents a compromise between early detection and ensuring sufficient data for robust prediction.

We added two sentences in the "Data preprocessing" section in "Materials and Methods" to clarify this:

> … week 28 includes weeks 0, 4, 12, and 28 data, etc.) The intervals between observation periods were prespecified in the structured protocols of the EXPEDITION trials from which our data were sourced. Such observation periods were designed with clinical relevance, as longer and later intervals are reflective of the expected gradual progression of AD. Hence, some timepoints were unavailable. We normalized …

5. ***The title of the article could have been more specific using the deep learning term instead of machine learning.***

We agree and changed the title to: "Deep learning to predict rapid progression of Alzheimer's disease from pooled clinical trials: A retrospective study."

6. ***For better understanding the model performance, the evaluation metric[s] like confusion matrix and F1 score can also be reported.***

Thank you for your suggestion. We reran the whole pipeline using 10-fold cross-validation and selected the classification thresholds for each fold to trade off the sensitivity and specificity on the validation set. Specifically, the thresholds were selected where sensitivity was larger than specificity for a margin of 0.15, ensuring a good sensitivity with a reasonable specificity. Then, we got 10 binary predictions on the hold-out test set based on the selected thresholds and the final binary predictions were determined by the majority over the 10 predictions for all the models. In this way, we calculated the confusion matrix and F1 score and reported them in the new Fig 3.

We also added the following content regarding confusion matrices and F1 scores in the "Evaluation" subsection in the "Materials and Methods" section:

> To further evaluate our model's binary prediction performances, we also displayed the confusion matrices and F1-scores for all models (Fig 3). The confusion matrix displays numbers of true negative (TN), false positive (FP), false negative (FN), and true positive (TP) in a 2×2 matrix, while the F1 score is the harmonic mean of precision and recall calculated as $F_1 = \frac{2 \cdot \text{precision} \cdot \text{recall}}{\text{precision} + \text{recall}} = \frac{2 \cdot \text{TP}}{2 \cdot \text{TP} + \text{FP} + \text{FN}}$. As we ran 10-fold cross-validation, we selected the classification thresholds for each fold to trade off the sensitivity and specificity on the validation set. Specifically, the thresholds were selected when sensitivity was larger than specificity with a margin ensuring a good sensitivity with a reasonable specificity (set to 0.15 in our case). Then, we had 10 binary predictions on the hold-out test set based on the selected thresholds. The final binary predictions were determined by the majority over the 10 predictions for all the models. The confusion matrices and F1 scores were computed based on the final predictions, with confusion matrices normalized over rows to show specificities in upper left boxes of matrices and sensitivities in lower right boxes of matrices (Fig 3).

We added this paragraph in the "Rapid progressor prediction" subsection in the "Results" section:

In addition, our models reached F1 scores ranging from 0.29 to 0.56 overall on the internal hold-out test set, while the models using data up to week 28 had F1 scores from 0.35 to 0.41 (Fig 3). The confusion matrices of 28-week models show sensitivities ranging from 0.74 to 0.88, with specificities ranging from 0.7 to 0.8, indicating good stratification between RPs and non-RPs.

The captions and plots of new Fig 3 is shown below:

Fig 3. Confusion matrices and F1 scores of RP prediction models using different definitions and observation periods.

Results include longitudinal models with internal validation. Confusion matrices are normalized over rows, and corresponding F1 scores are reported under the matrices. Each normalized confusion matrix displays specificity in the upper left box and sensitivity in the lower right box.

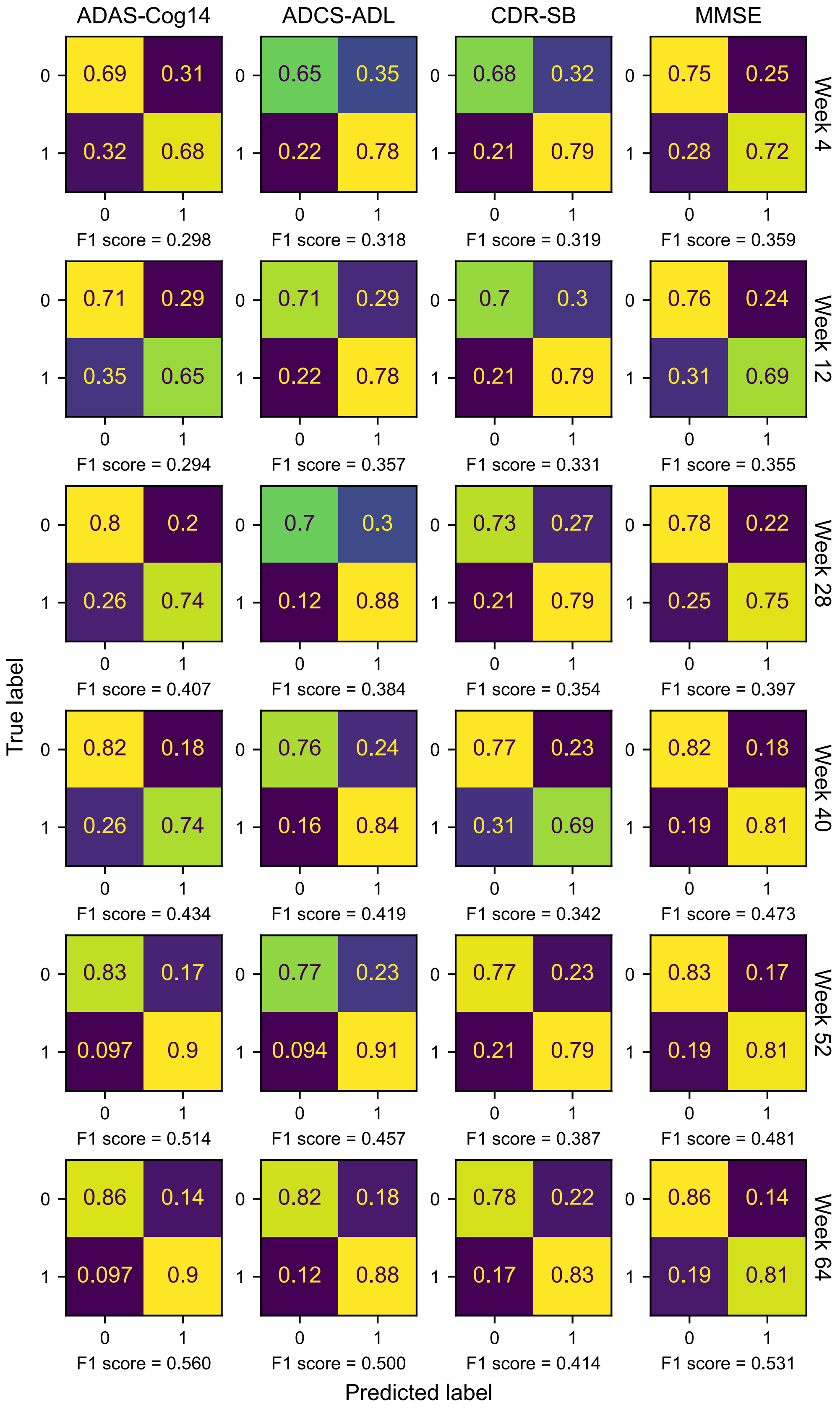

ADAS-Cog14
ADCS-ADL
CDR-SB
MMSE
Week 4
Week 12
Week 28
Week 40
Week 52
Week 64
True label
Predicted label
F1 score = 0.298
F1 score = 0.318
F1 score = 0.319
F1 score = 0.359
F1 score = 0.294
F1 score = 0.357
F1 score = 0.331
F1 score = 0.355
F1 score = 0.407
F1 score = 0.384
F1 score = 0.354
F1 score = 0.397
F1 score = 0.434
F1 score = 0.419
F1 score = 0.342
F1 score = 0.473
F1 score = 0.514
F1 score = 0.457
F1 score = 0.387
F1 score = 0.481
F1 score = 0.560
F1 score = 0.500
F1 score = 0.414
F1 score = 0.531

7. ***Most of the time (as can be seen in Figure 2) the differences in the accuracy of baseline model (2-layer MLP) and GRU model is not large, which can justify the added advantage of using GRU like model architecture with longitudinal data. It would be important to address if the same accuracy as the GRU model, can be achieved by optimizing baseline MLP model.***

We thank the reviewer for this constructive comment. To verify the significant accuracy gain, we reran the whole pipeline using 10-fold cross-validation and reported the confidence intervals. In the new Fig 2, we can see that the 28-week models still significantly outperform the baseline model in most cases (except the MMSE model with internal data split). Another point is the longitudinal GRU models use the same set of hyperparameters to fit on different periods of observations, while the MLP models only need to fit on the baseline data. As a result, there are a total of 24 longitudinal GRU models (6 observation periods and 4 labels) and only 4 baseline MLP models. This indicates that MLP models are more specifically optimized to the baseline data, while the GRU models are optimized to achieve generally good performances over all the time periods and labels. Even so, the GRU models trained on the 28-week observation periods can still significantly outperform the MLP models in most cases.

The captions and plots of new Fig 2 now reads:

> **Fig 2. AUROCs and AUPRCs of RP prediction models using different definitions and observation periods (including baselines).**
>
> Results include two evaluation modes: internal (to train and test with pooled data) and external (trained with EXPEDITION and EXPEDITION 2 and tested with EXPEDITION 3). We trained the models with 10-fold cross-validation and tested the trained models on the hold-out datasets for both evaluation settings, reporting mean AUROCs and AUPRCs over the 10 folds. The 95% confidence intervals were calculated by $1.96 \times \mathrm{SD}/\sqrt{n}$, where SD is the standard deviation over 10 folds and $n = 10$ is the number of folds.

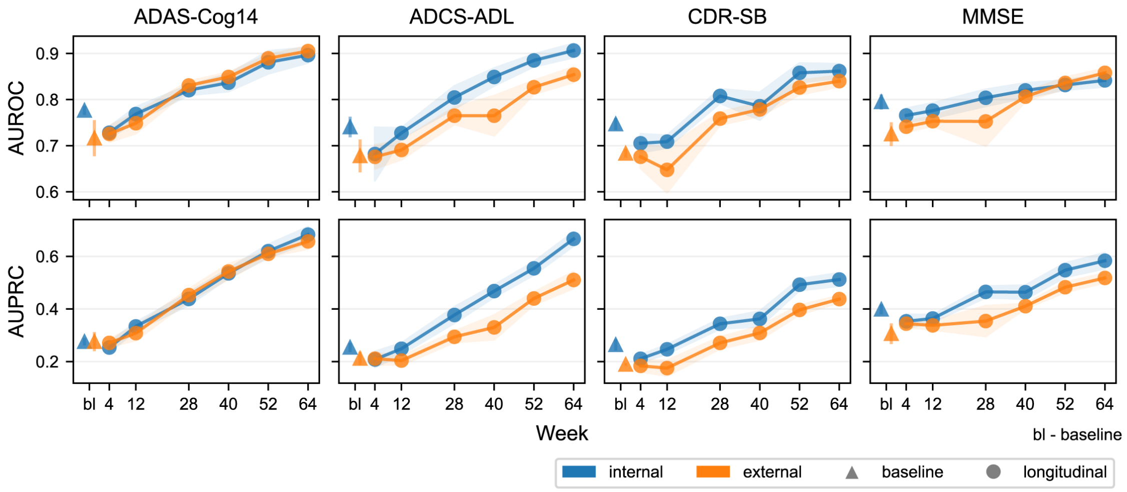

8. ***The manuscript Authors could do well by pointing out clear challenges in the analysis with respect to reproducibility on another dataset, potential confounders related to patient genetics beyond the standard markers such as ApoE (as AD is well known to have a genetic component) and how the current study could be improved in the future through models that may capture more variation.***

   We thank the reviewer and we do agree with this comment. It is possible that clinical trials may have a batch effect based on eligibility criteria and study sites. To mitigate reproducibility issues, we performed external validation (with data from EXPEDITION 3 as the external hold-out test set). Despite this, reproducibility remains challenging. Fortunately, the analysis we have used in our model is intended to capture components of standard protocols for clinical trials to ensure generalizability to other clinical trials' data. The main objective of this study was to develop a predictive model using the clinical trial gold-standard of available data—placebo arms—that can be extrapolated to data sources outside RCTs.

   We do acknowledge the prevalence of potential patient genetic components in AD, but seeing as there is little research into areas other than the standard marker such as ApoE (Liu & Li, 2019), we are using the information and the research available to us via the clinical trials to make the model as comprehensive as possible with the available data. There is the potential for the model to be tuned in the future to include and assess other genetic components that are predictive of a rapid progressor early on. To capture more variation of the data, we could include more cohorts of AD trials or clinical data (i.e., ADNI datasets) as our internal and external validation, as well as implement multi-modal multi-task deep learning models on different cohorts. Some more advanced deep learning architectures could be used to capture inherent variation of the tabular data, such as autoencoders and transformers.

   We added a few sentences in the limitation parts of the "Discussion" section to clarify this:

   > It is possible that clinical trials may have a batch effect based on eligibility criteria and study sites. To mitigate reproducibility issues, we performed external validation (with data from EXPEDITION 3 as the external hold-out test set). Fortunately, the analysis we have used in our model is intended to capture components of standard protocols for clinical trials in order to ensure generalizable to other clinical trials' data. The main objective of this study was to develop a predictive model using the clinical trial gold-standard of available data—placebo arms—that can be extrapolated to data sources outside RCTs.

   We also added two potential future directions in the "Discussion" section:

   > There is the potential for the model to be tuned in the future to include and assess other genetic components that are predictive of a rapid progressor early on [43,44].
   >
   > To capture more variations of the data, we could include more cohorts of AD trials or clinical data (i.e., ADNI datasets) as our internal and external validation, as well as implement multi-modal, multi-task deep learning models on different cohorts. Additionally, more advanced deep learning architectures, such as autoencoders and transformers, could be used to capture inherent variation of the tabular data.

   The citations are as follows:

43. Liu J, Li L. Targeting Autophagy for the Treatment of Alzheimer's Disease: Challenges and Opportunities. Front Mol Neurosci. 2019;12: 203.

44. Bekris LM, Yu C-E, Bird TD, Tsuang DW. Genetics of Alzheimer disease. J Geriatr Psychiatry Neurol. 2010;23: 213–227.

III. Response to Reviewer #3

1. ***With a sample size of 1603, 151 variables seem extreme.* Did the authors determine the sample size (DOI: 10.1002/sim.7992)?**

We appreciate the reviewer's comment that with a sample size of 1603, 151 variables appear to be a large number. We do agree with Reviewer #3 in that sufficient samples size are necessary to ensure the statistical power for multivariable prediction model, as referenced in the DOI: 10.1002/sim.7992. However, our approach is based on machine learning (specifically deep learning), rather than hypothesis testing in statistical models. Since there are no hypothesis tests, power analysis (thus calculating sample size) is not possible. Instead, we showed our model's confidence via cross validation and external validation.

In addition, we included many variables because the cause of AD is unclear; hence, many possible factors may contribute to its development. Therefore, we opted to include as many as relevant to the progression of both rapid and normal AD disease course.

We added this to the "Variables" subsection in the "Materials and Methods" section:

> Since the cause of AD is unclear and many possible factors may contribute to its development, we opted to include as many variables as relevant to predict RPs. We employed deep learning models to decide on significant variables instead of assuming a priori their importance.

2. ***The authors mentioned 64 baseline variables (22 demographics + 42 comorbid) and 87 longitudinal variables. I think these 87 longitudinal variables also have baseline measurements.***

Thank you for this comment. The longitudinal variables contain both baseline values and the subsequent longitudinal values. For the baseline model, we included both the 64 baseline variables, as well as the baseline measurements for the 87 longitudinal variables.

We added this to the "Variables" subsection in the "Materials and Methods" section:

> The longitudinal variables contained both baseline measurements as well as the subsequent measurements at later weeks (i.e., weeks 4, 12, 28, 40, 52, 64, and 80).

In addition, the sentence in the "Prediction model" subsection about the baseline models now reads:

> For comparison, we implemented simple two-layer multilayer perceptrons (MLPs) as a baseline model using only demographics, comorbidities, and baseline measurements for the longitudinal variables.

3. ***The definition of the RP needs proper citation. Why were the top 10% of patients with the largest score changes from baseline to week 80 classified as RPs? What's the basis of this 10% cut-off? In my opinion, a better definition would be to define RP as a time-to-event variable, e.g., time from the baseline to RP. The authors clearly ignore the time component, though they have access to rich datasets.***

   We appreciate the reviewer pointing this out. A current major challenge within the Alzheimer's disease community is the definition of a rapid progressor. While there are papers that discuss rapid progression of dementia patients, and some define rapid progressors within the context of their study, there is no universal definition.

   Furthermore, based on aducanumab trials and Biogen's input, the FDA decided to use a CDR-SB change of 8 points or more over 78 weeks for their definition. However, it is unclear how this definition was selected, much less how it was validated.

   To the best of our knowledge, our paper is the first to use four different cognitive assessments to define rapid progressors. We were able to validate all definitions with a gaussian mixture model (S1 Appendix, S1 Fig) where there is separation between patients who are rapid progressors and those who are not.

   For the last point, we aim to help such researchers in clinical trials make a better decision about enrollment criteria, and thus, we chose to predict RP as those researchers do. We have tried to mimic the actual primary endpoint analysis in clinical trials, where it is standard for researchers to investigate compare clinical outcomes at a fixed time point (e.g., 80 weeks). As our focus is to help in the refinement of clinical trials, we believe that our prediction target should resemble what researchers do in actual clinical trials, which is fixing a timepoint and predicting the clinical outcome at that time point. Finally, we make full use of the time component as an input (rather than label to predict).

   We added this to the "Definitions of a rapid progressor" subsection of the "Materials and Methods" section:

   > We defined rapid progressors (RPs) as the 10% of patients with the largest changes in cognitive scores from baseline to week 80. This was chosen empirically as clinical trialists have indicated that separating the fastest 10% of progressors would significantly benefit their trail design. We tested for RPs using these four cognitive measures: …

4. ***The data-splitting approach as an internal validation is a poor choice. The authors are advised to use better methods, such as cross-validation or bootstrapping (doi:10.1093/eurheartj/ehu207).***

   We appreciate the critique from the reviewer. Our original data splitting approach was indeed cross validation (70% training, 10% validation, 20% testing on discovery set). Nonetheless, to make a more robust evaluation, we reran our whole pipeline using 10-fold cross validation on both internal and external validation. We used the same hold-out test set for both internal and external validation as before, and further split the remaining samples into 10 folds. In the 10-fold cross-validation, we

reserved 1-fold for validation to select the best models and used the remaining 9 folds for training each time. All the folds were split on the subject level to avoid data leakage. In this way, we had 10 checkpoints corresponding to 10 folds and calculated the means and standard deviations (SDs) of AUROC and AUPRC over the 10 folds. The final 95% confidence intervals are calculated by $1.96 \times SD/\sqrt{n}1.96 \times SD/\sqrt{n}$, where $n = 10$ indicates the number of folds in this case. We believe the 10-fold cross validation could better evaluate the performances of our models.

We added the description of 10-fold cross-validation in the "Evaluation" subsection in the "Materials and Methods" section:

> We evaluated the models using internal and external hold-out validation combined with 10-fold cross-validation. We split the pooled data (n = 1603) into a training set for 10-fold cross-validation (n = 1282, 80%) and a hold-out test set (n = 321, 20%). For external validation, we set aside the EXPEDITION 3 trial as the hold-out test set (n = 844), considering its different inclusion criteria, and treated the pooled EXPEDITION and EXPEDITION 2 trials (n = 759) as the training set for 10-fold cross validation. We then split the training sets in both internal and external validation into 10 folds, reserving 1-fold for validation to select the best models and using the remaining 9 folds for model training each time.
>
> We assessed prediction accuracy using the area under the receiver operating characteristic curve (AUROC) and the area under the precision and recall curve (AUPRC) (Fig 2). The AUROC measures the probability that the predicted scores for the ground-truth positive samples are larger than those of the negative samples, with 0.5 as the baseline referring to a random classifier. The AUPRC measures the trade-off between precision (fraction of true positive among predicted positive) and recall (fraction of true positive among ground-truth positive), with a higher AUPRC indicating the model can identify most of the positive samples without classifying too many negative samples as positive. The baseline value for AUPRC is the positive rate of the data, approximately 10% in our case. We reported the averaged AUROCs and AUPRCs over 10 folds for all trained models and calculated their 95% confidence intervals via $1.96 \times \mathrm{SD}/\sqrt{n}$, where SD is the standard deviation of a metric over 10 folds, and $n = 10$ is the number of folds.

We also reported the updated model accuracy in the new Fig 2:

> **Fig 2. AUROCs and AUPRCs of RP prediction models using different definitions and observation periods (including baselines).**
>
> Results include two evaluation modes: internal (to train and test with pooled data) and external (trained with EXPEDITION and EXPEDITION 2 and tested with EXPEDITION 3). We trained the models with 10-fold cross-validation and tested the trained models on the hold-out datasets for both evaluation settings and reported mean AUROCs and AUPRCs over the 10 folds. The 95% confidence intervals were calculated by $1.96 \times \mathrm{SD}/\sqrt{n}$, where SD is the standard deviation over 10 folds and $n = 10$ is the number of folds.

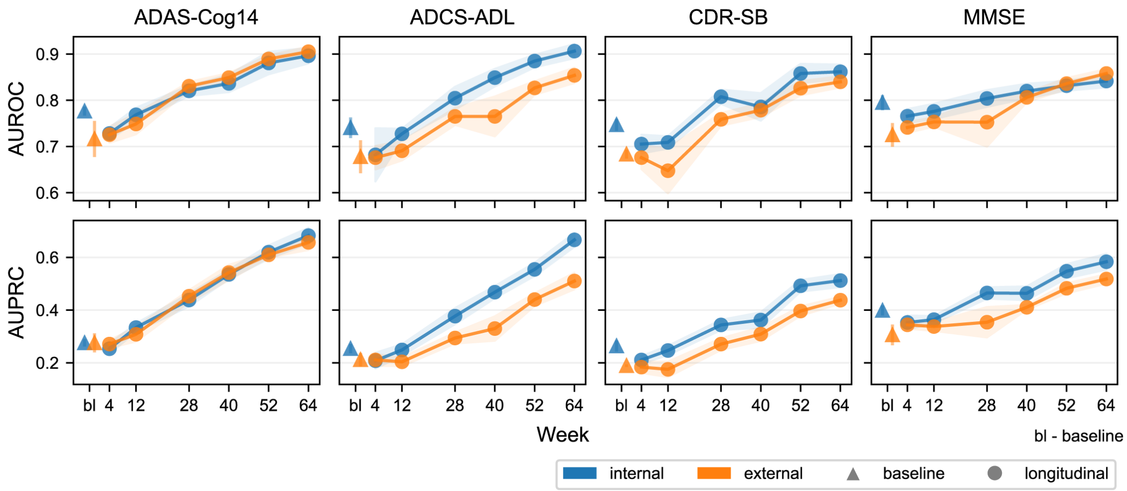

5. ***Since the authors utilized the longitudinal data, I am surprised that the authors report the time-invariant AUC instead of the time-dependent AUC. However, in addition to the AUC, the authors should report the calibration (intercept and slope) and the model's overall performance (e.g., brier score). A decision-curve analysis is also recommended. (doi:10.1093/eurheartj/ehu207)***

We appreciate the comprehensive statistical analysis suggested for evaluation of our models. It is possible, however, that the time-dependent AUC may not be suitable for our situation as we do not have time-dependent labels as one would see in a survival model. Instead, we have pre-defined binary labels of rapid progression at week 80. Our models use different time periods from baseline to predict the binary label, and we do not have labels for each time point. The calibration curve, brier score, and the decision-curve analysis evaluate whether prediction models can output accurate risk probabilities. However, in our scenario, the purpose of our models is not to give accurate risk probability to a certain event (e.g., death), but to stratify the patients into two groups with potentially different patterns of progression, i.e., RP and non-RP, for better clinical trial design.

The RP and non-RP themselves also do not have certain distinctions, so we investigated several potential definitions of RP based on our cohorts. We used 10-fold cross-validation to get 10 candidate models outputting logits, which are then used to predict the binary RP labels using the selected thresholds of logits on corresponding validation sets. In this way, we obtained 10 predicted binary labels, and the final predicted label was determined by the majority of the 10 labels. Otherwise, if we directly use the output logits to calculate probabilities for each fold, the models will lean to predicting non-RPs even if the "probabilities" are high, which would not show the real performances of our models. As a result, we reported the confusion matrices and the F1-scores based on our final predictions after majority voting on 10 folds (new Fig 3), as well as the mean AUROCs and AUPRCs over the 10 folds (new Fig 2). We would please suggest that these are more suitable metrics to evaluate our models.

We added the description of 10-fold cross-validation in the "Evaluation" subsection in the "Materials and Methods" section:

To further evaluate our model's binary prediction performances, we also displayed the confusion matrices and F1-scores for all models (Fig 3). The confusion matrix displays numbers of true negative (TN), false positive (FP), false negative (FN), and true positive (TP) in a 2×2 matrix, while the F1 score is the harmonic mean of precision and recall calculated as $F_1 = \frac{2 \cdot \text{precision} \cdot \text{recall}}{\text{precision} + \text{recall}} = \frac{2 \cdot \text{TP}}{2 \cdot \text{TP} + \text{FP} + \text{FN}}$. As we ran 10-fold cross-validation, we selected the classification thresholds for each fold to trade off the sensitivity and specificity on the validation set. Specifically, the thresholds were selected when sensitivity was larger than specificity with a margin ensuring a good sensitivity with a reasonable specificity (set to 0.15 in our case). Then, we had 10 binary predictions on the hold-out test set based on the selected thresholds. The final binary predictions were determined by the majority over the 10 predictions for all the models. The confusion matrices and F1 scores were computed based on the final predictions, with confusion matrices normalized over rows to show specificities in upper left boxes of matrices and sensitivities in lower right boxes of matrices (Fig 3).

We added this paragraph in the "Rapid progressor prediction" subsection in the "Results" section:

In addition, our models reached F1 scores ranging from 0.29 to 0.56 overall on the internal hold-out test set, while the models using data up to week 28 had F1 scores from 0.35 to 0.41 (Fig 3). The confusion matrices of 28-week models show sensitivities ranging from 0.74 to 0.88, with specificities ranging from 0.7 to 0.8, indicating good stratification between RPs and non-RPs.

The confusion matrix and F1 scores are reported in the new Fig 3:

Fig 3. Confusion matrices and F1 scores of RP prediction models using different definitions and observation periods.

Results include longitudinal models with internal validation. Confusion matrices are normalized over rows, and corresponding F1 scores are reported under the matrices. Each normalized confusion matrix displays specificity in the upper left box and sensitivity in the lower right box.

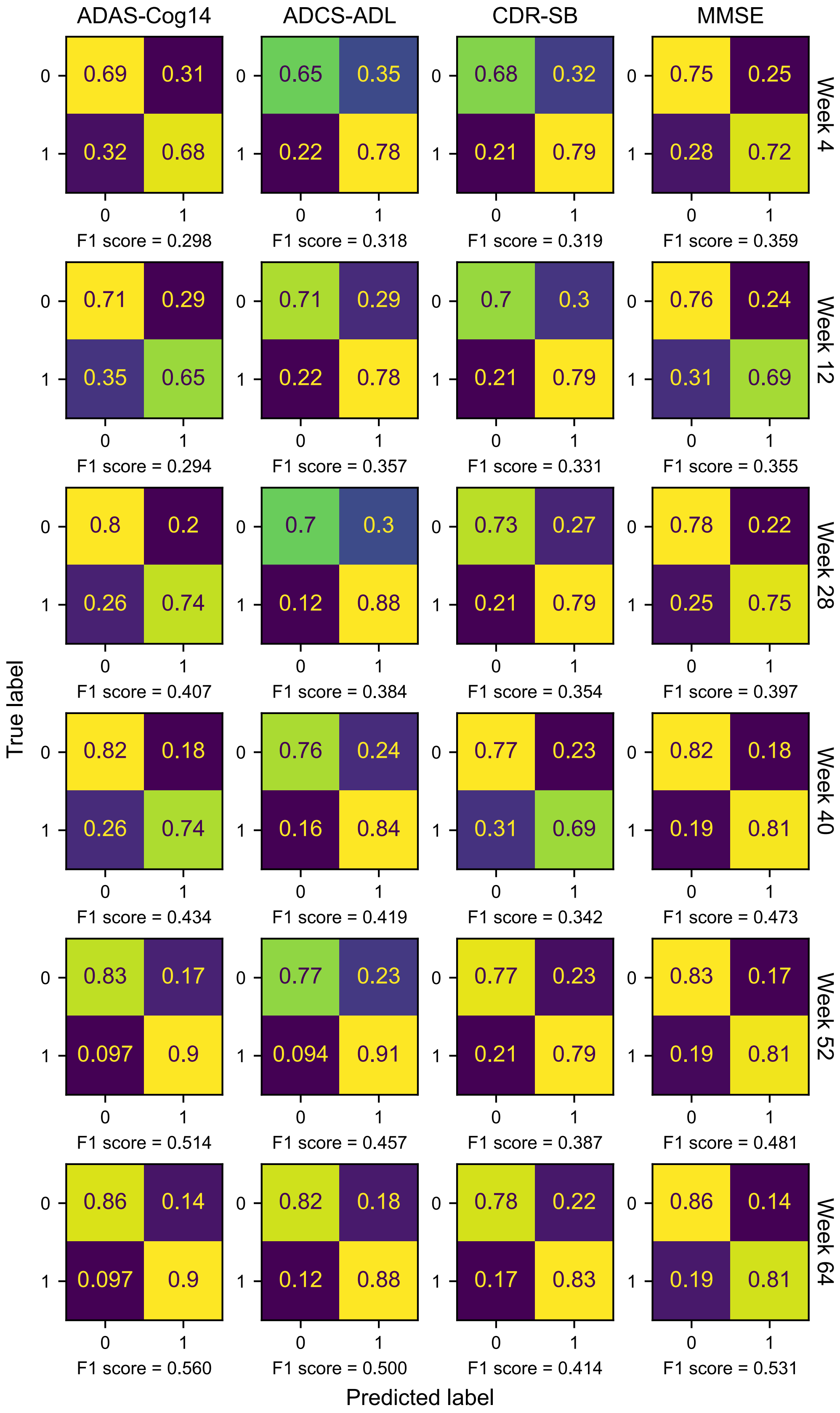
ADAS-Cog14
ADCS-ADL
CDR-SB
MMSE
Week 4
Week 12
Week 28
Week 40
Week 52
Week 64
True label
Predicted label
0.69 0.31 0.32 0.68
0.65 0.35 0.22 0.78
0.68 0.32 0.21 0.79
0.75 0.25 0.28 0.72
F1 score = 0.298
F1 score = 0.318
F1 score = 0.319
F1 score = 0.359
0.71 0.29 0.35 0.65
0.71 0.29 0.22 0.78
0.7 0.3 0.21 0.79
0.76 0.24 0.31 0.69
F1 score = 0.294
F1 score = 0.357
F1 score = 0.331
F1 score = 0.355
0.8 0.2 0.26 0.74
0.7 0.3 0.12 0.88
0.73 0.27 0.21 0.79
0.78 0.22 0.25 0.75
F1 score = 0.407
F1 score = 0.384
F1 score = 0.354
F1 score = 0.397
0.82 0.18 0.26 0.74
0.76 0.24 0.16 0.84
0.77 0.23 0.31 0.69
0.82 0.18 0.19 0.81
F1 score = 0.434
F1 score = 0.419
F1 score = 0.342
F1 score = 0.473
0.83 0.17 0.097 0.9
0.77 0.23 0.094 0.91
0.77 0.23 0.21 0.79
0.83 0.17 0.19 0.81
F1 score = 0.514
F1 score = 0.457
F1 score = 0.387
F1 score = 0.481
0.86 0.14 0.097 0.9
0.82 0.18 0.12 0.88
0.78 0.22 0.17 0.83
0.86 0.14 0.19 0.81
F1 score = 0.560
F1 score = 0.500
F1 score = 0.414
F1 score = 0.531

6. ***The higher AUC from the model with longitudinal data is expected from a statistical point of view. However, with longitudinal data, it is unclear why the authors did not consider a dynamic prediction model that can be updated over time (DOI: 10.1111/rssa.12611).***

Thank you for your comment. To clarify, our model took the longitudinal data into account in the GRU model, and we showed the higher AUC due to the longitudinal data compared to the one without longitudinal data in Fig. 2. Regarding the referenced paper, dynamic prediction models predict the survival on some future horizon time points t_{hor} conditioned on variables measured up to a certain time point s, where $s < t_{hor}$. It is a useful technique for survival analysis enabling updating the prediction based on new measurements at new times. However, we are not performing survival analysis since we did not have event time and censoring time for rapid progression. On the other hand, the RPs themselves are defined as binary label on the fixed time point of week 80 by predetermined thresholds, i.e., if the changes of scores from baseline to week 80 are greater than the predefined threshold. It is an inherently different design compared to a survival analysis, which would define RP as an event which is not related to time itself (e.g., death, disease recurrence, ICU admission, etc.), then checking the event time and censoring time for each patient. Nonetheless, the reviewer's suggestion could open a new study direction of AD rapid progressors, formatting the problem into a survival analysis framework.

We appreciate the reviewer's insights and have added this in the future works in "Discussion" section:

> Survival analysis could be explored by treating RP as a time-to-event variable, implementing a dynamic prediction model that can be updated over time [38], and evaluating the models by time-dependent AUROC.

Citation:

> 38. Tanner KT, Sharples LD, Daniel RM, Keogh RH. Dynamic Survival Prediction Combining Landmarking with a Machine Learning Ensemble: Methodology and Empirical Comparison. J R Stat Soc Ser A Stat Soc. 2021;184: 3–30.

7. ***More than 30% of missing data seems high. The authors used multiple imputation, which is good. But it is unclear how they evaluate the model performance with multiple imputation. Also, did the authors conduct a sensitivity analysis with a different assumption than missing at random?***

We thank the reviewer for this comment and would like to note the added evaluation of several imputation strategies. Our data consist of baseline variables and longitudinal features. The baseline variables include patient demographics, comorbidities, and other neurocognitive measurements. The comorbidities are binary variables, and we only included those with ≤ 7 missing values out of 1603 samples (missing rate ~ 0.44%), which is small enough to legitimately impute the missing values as 0 (no existence) since it is the major class for all comorbidities. Then, for the remaining numerical baseline variables, we used two imputation strategies: (1) simple imputation with mean values of each column, which assumes the missing to be MCAR; (2) multiple imputation by chained

equation (MICE), which assumes the missing pattern to be MAR. For longitudinal variables, the missing values are mainly certain observations for some time steps in each patient.

We further compared three imputation methods: (1) last observation carried forward (LOCF), assuming that the longitudinal data has temporal stability as the cohorts we used are placebo arms of clinical trials; (2) linear imputation, which assumes temporal linearity between observed time steps; and, (3) MICE, which assumes the missing pattern to be MAR and depend on other observed values. For longitudinal imputation (2) and (3), we carefully avoided the temporal data leakage by imputing data within the observed periods according to the specific observation periods for each model. Thus, we had 6 combinations of imputation methods between baseline and longitudinal variables. The 6 imputation strategies were evaluated by the internal split and 10-fold cross-validation. The AUROC and AUPRC plots are reported in the appendix. We found that the two imputation strategies for baseline variables do not show significant difference, while the MICE imputation for longitudinal variables showed marginal improvement. As a result, we updated our imputation strategies by using MICE for both baseline and longitudinal variables. Furthermore, we proposed potential future works on data imputation to improve our models in the "future works" section.

We reported our experiments on imputation evaluation in the supporting information S1 Appendix "Imputation strategies" section:

> **Imputation strategies**
>
> We tried several imputation strategies to deal with missing data in our baseline variables and longitudinal variables. The baseline variables include patient demographics, comorbidities, and other neurocognitive measurements. The comorbidities are binary variables, and we only included those with ≤ 7 missing values out of 1603 samples (missing rate ~ 0.44%), which is small enough to legitimately impute the missing values as 0 (no existence) since it is the major class for all comorbidities. Then, for the remaining numerical baseline variables, we used two imputation strategies: (1) simple imputation with mean values of each column, which assumes the missing to be MCAR; and, (2) multiple imputation by chained equation (MICE), which assumes the missing to be MAR. For longitudinal variables, the missing values are mainly certain observations for some time steps in each patient.
>
> We further compared three imputation methods: (1) last observation carried forward (LOCF), assuming that the longitudinal data has temporal stability as the cohorts we used are placebo arms of clinical trials; (2) linear imputation, which assumes temporal linearity between observed time steps; (3) MICE, which assumes the missing to be MAR and depend on other observed values. For longitudinal imputation (2) and (3), we carefully avoided the temporal data leakage by imputing data within the observed periods according to the specific observation periods for each model. Thus, we have 6 combinations of imputation methods between baseline and longitudinal variables. The 6 imputation strategies were evaluated by the internal split and 10-fold cross-validation. The AUROC and AUPRC plots are reported in the appendix. We found that the two imputation strategies for baseline variables do not show significant difference, while the MICE imputation for longitudinal variables showed marginal improvement. As a result, we updated our imputation strategies by using MICE for both baseline and longitudinal variables.

We also added S2 Fig to show AUROCs and AUPRCs of RP prediction models of different imputation strategies:

S2 Fig. AUROCs and AUPRCs of RP prediction models of different imputation strategies.

(A) Baseline variables: mean imputation. Longitudinal variables: 1) last observation carried forward (LOCF); 2) linear imputation; and, 3) multiple imputation by chained equation (MICE). (B) Baseline variables: MICE. Longitudinal variables: 1) LOCF; 2) linear imputation; and, 3) MICE. (C) Baseline variables: 1) mean imputation; and, 2) MICE. Longitudinal variables: MICE.

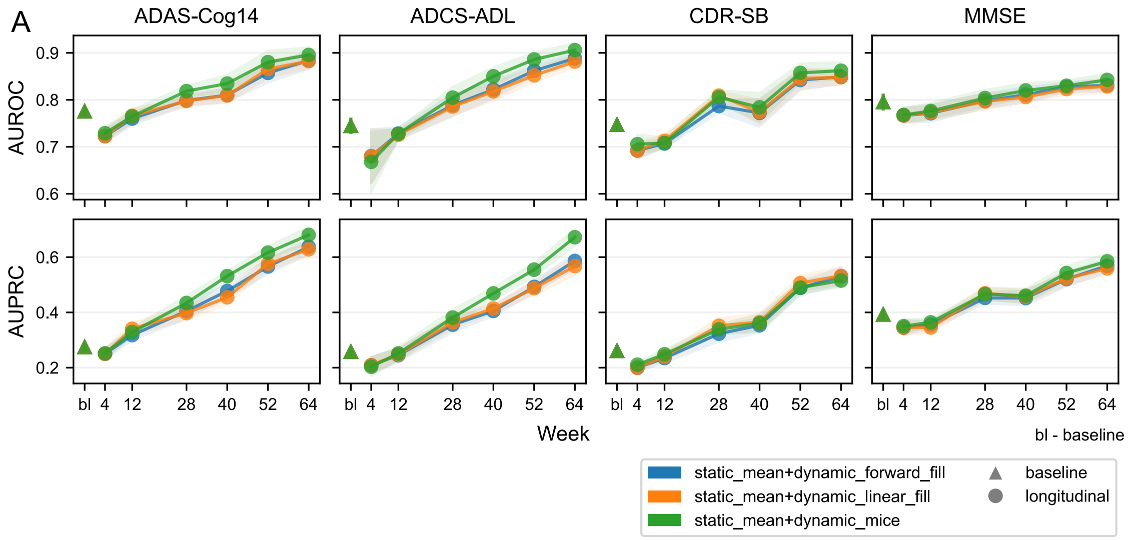

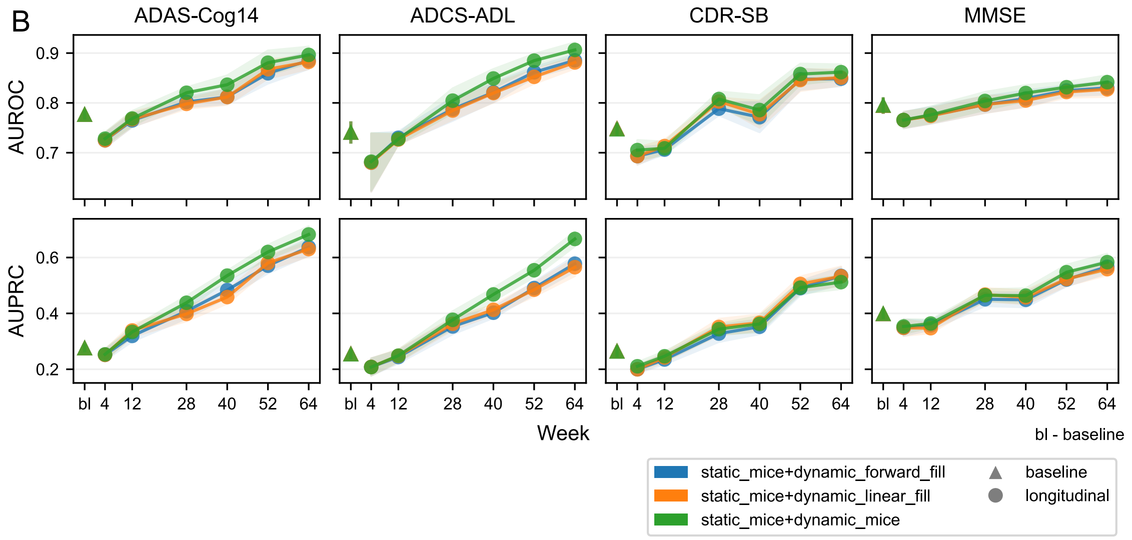

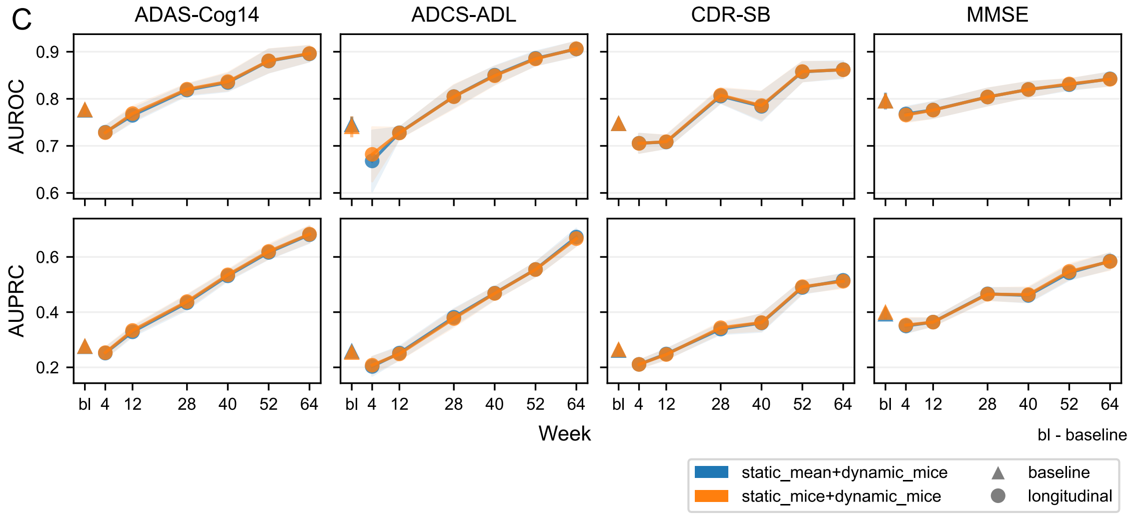

In the main text, the imputation part of "Data preprocessing" section in "Materials and Methods" now reads:

> Missing values in longitudinal variables were also imputed by MICE, assuming missing at random. We carefully avoided the temporal data leakage by imputing data within the observed periods according to the specific observation periods for each model. S1 Appendix and S2 Fig show details regarding how we evaluate and select from several imputation strategies.

We also proposed one future work in the "Discussion" section:

> More imputation methods could be explored on our baseline and longitudinal data, such as the data-driven missing value imputation approach [39], HyperImpute [40], or integrating the imputation module into the prediction models [41].

The citations here are:

39. Ribeiro C, Freitas AA. A data-driven missing value imputation approach for longitudinal datasets. Artificial Intelligence Review. 2021;54: 6277–6307.

40. Jarrett D, Cebere BC, Liu T, Curth A, van der Schaar M. HyperImpute: Generalized Iterative Imputation with Automatic Model Selection. In: Chaudhuri K, Jegelka S, Song L, Szepesvari C, Niu G, Sabato S, editors. Proceedings of the 39th International Conference on Machine Learning. PMLR; 17--23 Jul 2022. pp. 9916–9937.

41. Xu L, Wu H, He C, Wang J, Zhang C, Nie F, et al. Multi-modal sequence learning for Alzheimer's disease progression prediction with incomplete variable-length longitudinal data. Med Image Anal. 2022;82: 102643.

8. ***It is unclear why the authors dichotomized the age variable. And some categories should be merged due to small frequencies (e.g., race, APOE type).***

We thank the reviewer for this comment regarding variable selection.

(1) Age variables. We give four different age definitions in Table 1. We chose to dichotomize the age into less than 65 years old, and 65 and older because this is an important distinction within Alzheimer's disease. If a patient is less than 65 years old when they are diagnosed, then it is considered to be early onset Alzheimer's, and for those 65 years and older, it is termed late onset AD. Readers, clinicians or otherwise, who read our manuscript for the implications regarding Alzheimer's disease will be interested in this information. During model training, however, we still use the continuous age numbers for AD onset and exact ages.

(2) Categories with small frequencies (e.g., race, APOE type). There is an increasingly evident divide in AD presentation between races and in predisposition by genotype (Liu & Li, 2019; Bekris et al., 2011). Therefore, we believe that it is highly relevant, both clinically and computationally, to preserve these less-frequent variables in the prediction model(s).

References:

Liu J, Li L. Targeting Autophagy for the Treatment of Alzheimer's Disease: Challenges and Opportunities. Front Mol Neurosci. 2019;12: 203.

Bekris LM, Yu C-E, Bird TD, Tsuang DW. Genetics of Alzheimer disease. J Geriatr Psychiatry Neurol. 2010;23: 213–227.

9. ***Overall, whether the models used can select spare a model is unclear. The clinicians may not be interested in using this prediction model because of the large number of predictors with a black box model. There should be a justification for why clinicians could be interested in the model the authors had developed.***

We appreciate this comment and understand the reviewer's concern with the accessibility of the model for clinicians. Here we would like to emphasize the importance of prediction accuracy before the accessibility. AD is a very complex and multifaceted disease, and therefore it requires a multitude of variables to diagnose, including the results of cognitive testing, MRIs, ApoE Genotyping, PET Scans, and/or lumbar punctures. In order to have a comprehensive and definitive diagnosis, these variables are necessary for an AD diagnosis. Additionally, the variables used in our model are part of the standard protocols for clinical trials, including the clinical trials from which our database is derived (EXPEDITION, EXPEDITION 2, EXPEDITION 3), making the model generalizable to other clinical trials' data.

The main objective of this study was to develop a predictive model using the clinical trial gold-standard of available data, placebo arms, to lay the groundwork for developing an accurate prediction model that can be extrapolated to data sources outside RCTs. Our results will lead to more targeted and effective clinical trials by allowing investigators to better select trial participants that are more likely to experience rapid progression. The next steps for our project include using clinical data to replicate our model and our findings. With the continued refinement of both our definition of a RP and our prediction model, we aim to provide a tool with which clinicians can better predict, treat, and serve patients with rapidly progressing AD and their families.

We revised the conclusion in the new "Conclusion" section to emphasize the promising findings and applications of our models:

> Our research represents a breakthrough in predicting the rapid progression of AD. By analyzing pooled placebo-arm data from three randomized controlled trials, we developed advanced deep learning models capable of accurately identifying rapidly progressive AD cases.
>
> A critical discovery of our research was the identification of key biomarkers and neurocognitive health indicators that are instrumental in predicting RPs. These include the plasma level $A\beta_{1-42}/A\beta_{1-40}$ ratio, regional brain volumetry, and specific areas of cognitive decline. These findings are important in the context of AD therapy development, as they might empower researchers to design clinical trials that more effectively account for the diverse ways in which AD symptoms can manifest and progress in patients.
>
> In summary, our research represents a significant advancement in understanding and predicting the rapid progression of AD. The utilization of deep learning models, derived from comprehensive data across multiple trials, has enabled us to identify crucial factors in AD progression. This enhances our ability to predict which patients are more likely to experience rapid progression. The implications of these findings are profound, paving the way for more targeted and effective clinical trials. This, in turn, promotes the development of AD therapies that are better suited to address the varied trajectories of symptom progression in patients, ultimately leading to more effective treatment strategies for this complex and challenging condition.

10. ***The authors should report the guideline they followed in developing the prediction model.***

    We thank the reviewer for this comment, but we are unclear whether the reviewer is referencing the guideline for our RP definition or development of the specific deep learning model. In the event it is the former, we invite the reviewer to see our previous response to the second reviewer's third comment. Seeing as there is no concrete definition of a RP in AD, we used the marginal threshold of top or bottom 10% of scores on each of our four neurocognitive testing measures before empirically validating these thresholds with a gaussian mixture model. In the event it is the latter, we followed the empirical evidence of machine learning research to use recurrent neural network (gated recurrent unit in our cases) to model the longitudinal data. For the discussion of attention mechanisms, please refer to our response to Review #4 Comment 1.

IV. Response to Reviewer #4

1. ***Clarifying the Role of the Attention Mechanism: The analytical method utilised in the paper was a recurrent neural network architecture with an additional attention mechanism module. This attention mechanism, as mentioned, is a great standard to use in longitudinal data classification. As you mentioned, it can focus on temporal dependency by, for instance, favouring the most recent time steps information over the older ones, assuming that the patient's status of the most recent time steps is most likely closer than that of the older time steps. As a machine learning***

applied medicine individual, I do comprehend these concepts; however, a portion of the journal's readers are likely to have a strong medical background and may be unfamiliar with neural networks and attention mechanisms. While neural network literature and materials are readily available, I believe that attention mechanisms in the context of longitudinal data are at the very least more specialised. Therefore, I would strongly appreciate it if you considered a solution in this direction, if at all feasible. E.g., An appendix enhancement could include a brief subsection describing how attention mechanisms function and how they could be utilised in networks to prioritise more recent longitudinal data over older data. Another option could be a straightforward citation to a paper or survey or something similar that explains it well for any reader who may be interested in applying it to their medical-based work; however, I believe this should be directed towards the attention mechanism for longitudinal data. I would strongly recommend assisting the readers in comprehending the purpose of the attention mechanism in this longitudinal's context, utilizing one of the aforementioned proposed solutions or one you deem to be of greater value.

We really appreciate this constructive suggestion. We visualized the mean attention heatmaps over subjects in the text set averaging across the 10 folds. The results showed that the attention module tried to focus more on some specific time steps than the others, and the patterns were different across labels and observation periods. For example, for the MMSE label, the attention weights of earlier time points are higher, while for the ADAS-Cog14 and ADCS-ADL labels, later time points weigh more. Future works will investigate the displayed attention patterns for different labels or combinations of labels.

We added details in supporting information discussing the attention mechanisms. In S1 Appendix, we added a subsection "Attention weights heatmaps" about how we obtained the attention heatmaps for all the models and described the results:

> **Attention weight heatmaps**
>
> We visualized the attention weights for all models as heatmaps, where yellow indicates high attention while blue indicates low attention. For a specific model trained on N observations, the attention weights are $A = [a_1, a_2, \dots a_N]$ where a_n is calculated as described above (in "Rapid progression prediction model" section). S3 Fig shows the attention weights averaged over 10 folds for all the models, and some patterns can be summarized: (1) when predicting labels defined by ADAS-Cog14 and ADCS-ADL, later time point had higher importance for model prediction; (2) when predicting labels defined by MMSE, earlier time points contributed more to the model prediction; (3) when predicting labels defined by CDR-SB, the attention maps did not show common patterns across different observation periods. However, the clinical insights of these patterns are not clear and further research could investigate these findings.

We have also added S3 Fig in supporting information to show the obtained attention heatmaps, which are averaged over 10 folds on observed time steps for all trained models:

> **S3 Fig. Attention heat maps on observed time steps for all models.**

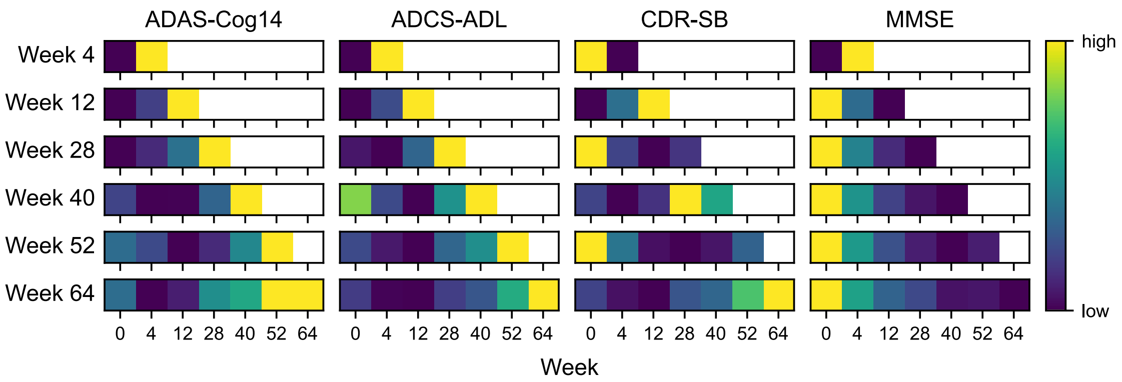

2. ***Motive Behind Metric Selection: The metrics of interest in the paper are AUROC and AUPRC. Both are highly utilised analytical metrics in academia and industry. In a medical context, however, when data are imbalanced, AUPRC tends to be more valued than AUROC, despite the fact that there are other motives for this as well. In the meantime, I would encourage the authors to elaborate on their motives for selecting these two specific metrics. While it is acknowledged that these metrics are frequently used in medical contexts, it is unclear whether the data under consideration are imbalanced or if other cogent arguments favour their selection. Therefore, to avoid any potential ambiguity, I suggest that the authors either elaborate on this in the main body of the manuscript or in an appendix.***

We thank the reviewer for this insightful comment. We selected AUROC and AUPRC as our performance metric since our dataset is imbalanced; we defined the label(s) using 10% quantiles as threshold, so the positive rate is expected to be 10%. The AUROC measures the probability that the predicted scores (logits in our case) of the positive samples are larger than the predicted scores of the negative samples. The baseline for AUROC is 0.5, which is referring to a random classifier for binary classification. The AUPRC calculates the trade-off between precision (the fraction of true positive among predicted positive samples) and recall (the fraction of true positive among ground-truth positive samples) across different thresholds. A high AUPRC score indicates that the model can identify most of the positive samples without classifying too many negative samples as positive. The baseline value for AUPRC is the positive rate in the dataset, which is approximately 10% in our cases. As a result, our model achieved promising results in both AUROC and AUPRC, demonstrating good prediction performance.

In addition, to further evaluate our model's binary prediction performances, we add the confusion matrix and F1-score as our metrics. As we ran 10-fold cross-validation, we selected the best decision threshold for each fold on the corresponding validation set and the final binary prediction was determined by the majority of the 10 predictions on the test set.

3. ***Justification for Evaluation Methodology: The chosen evaluation method, which could be as a hold-out external set validation method, is extremely advantageous in certain circumstances. However, it is simple to engage in the discussion, as a reader, why K-Fold Cross Validation has not***

been employed instead. While statistically, these evaluation methodologies have advantages and disadvantages, I believe there is a reason you chose the holdout-based validation method over others. For instance, a straightforward reason could be that this method is more computationally and timely efficient, whereas you may not have had both resources to execute K-fold cross-validation during your study. Another reason I can think of is that standard K-fold cross-validation could result in longitudinal data leakage, which is not necessarily representative of the real-world scenario. However, this is contingent upon the data structure of your longitudinal characteristics, to which I did not have access. Due to the longitudinal characteristics of each patient that manifest across the row direction of a dataset, K-fold cross validation can inadvertently introduce data leakage by splitting information from the same patients, making the method less reliable for real-world scenarios. Consequently, the hold-out external set serves as an excellent alternative for evaluation, as it circumvents the risk of this longitudinal data leakage. As a result, as a reader, it was unclear why this evaluation method was chosen and for what specific reasons. Hence, it would be greatly appreciated if the manuscript or appendix provided the actual explanation. Again, we believe that you may have additional, potentially superior solutions, so please feel free to act accordingly.

We thank the reviewer for the comments on model evaluation method. To more robustly evaluate our models' performances, we updated the model evaluation using 10-fold cross-validation with a hold-out test set for both internal and external validation and reran our whole pipeline. We used the same internal and external hold-out test sets as before, and further split the remaining samples into 10 folds. In the 10-fold cross-validation, we reserved 1-fold for validation to select the best models and used the remaining 9 folds for training each time. All the folds were split on the subject level to avoid data leakage. We had 10 checkpoints corresponding to 10 folds and calculated the means and standard deviations (SDs) of AUROC and AUPRC over the 10 folds. The final 95% confidence intervals are calculated by $1.96 \times \mathrm{SD}/\sqrt{n}$, where $n = 10$ indicates the number of folds in this case. We believe the 10-fold cross-validation with a hold-out test set could better evaluate the performances of our models when generalizing to new data in real-world scenarios by taking both advantages of hold-out validation and K-fold cross-validation.

Considering the above two comments, the "Evaluation" subsection of the "Materials and Methods" section was rewritten as the following:

> We evaluated the models using internal and external hold-out validation combined with 10-fold cross-validation. We split the pooled data (n = 1603) into a training set for 10-fold cross-validation (n = 1282, 80%) and a hold-out test set (n = 321, 20%). For external validation, we set aside the EXPEDITION 3 trial as the hold-out test set (n = 844), considering its different inclusion criteria, and treated the pooled EXPEDITION and EXPEDITION 2 trials (n = 759) as the training set for 10-fold cross validation. We then split the training sets in both internal and external validation into 10 folds, reserving 1-fold for validation to select the best models and using the remaining 9 folds for model training each time.
>
> We assessed prediction accuracy using the area under the receiver operating characteristic curve (AUROC) and the area under the precision and recall curve (AUPRC) (Fig 2). The AUROC

measures the probability that the predicted scores for the ground-truth positive samples are larger than those of the negative samples, with 0.5 as the baseline referring to a random classifier. The AUPRC measures the trade-off between precision (fraction of true positive among predicted positive) and recall (fraction of true positive among ground-truth positive), with a higher AUPRC indicating the model can identify most of the positive samples without classifying too many negative samples as positive. The baseline value for AUPRC is the positive rate of the data, approximately 10% in our case. We reported the averaged AUROCs and AUPRCs over 10 folds for all trained models and calculated their 95% confidence intervals via $1.96 \times \mathrm{SD}/\sqrt{n}$, where SD is the standard deviation of a metric over 10 folds, and $n = 10$ is the number of folds.

To further evaluate our model's binary prediction performances, we also displayed the confusion matrices and F1-scores for all models (Fig 3). The confusion matrix displays numbers of true negative (TN), false positive (FP), false negative (FN), and true positive (TP) in a 2×2 matrix, while the F1 score is the harmonic mean of precision and recall calculated as $F_1 = \frac{2 \cdot \mathrm{precision} \cdot \mathrm{recall}}{\mathrm{precision} + \mathrm{recall}} = \frac{2 \cdot \mathrm{TP}}{2 \cdot \mathrm{TP} + \mathrm{FP} + \mathrm{FN}}$. As we ran 10-fold cross-validation, we selected the classification thresholds for each fold to trade off the sensitivity and specificity on the validation set. Specifically, the thresholds were selected when sensitivity was larger than specificity with a margin ensuring a good sensitivity with a reasonable specificity (set to 0.15 in our case). Then, we had 10 binary predictions on the hold-out test set based on the selected thresholds. The final binary predictions were determined by the majority over the 10 predictions for all the models. The confusion matrices and F1 scores were computed based on the final predictions, with confusion matrices normalized over rows to show specificities in upper left boxes of matrices and sensitivities in lower right boxes of matrices (Fig 3).

4. ***Considerations for Missing Value Imputations: While preprocessing techniques have been investigated, carrying forward the last observation remains a frequently used data preprocessing technique for missing values. There are many more statistical missing value imputation methods, as well as supervised machine learning methods and data-driven methods for missing value imputation, specifically applicable to longitudinal datasets. While, furthermore, the labour-intensive nature of the exploration is the best for a specific scenario, focused-missing value imputation systems utilising Bayesian optimisation or even a brute-force or random search for finding the best setting for your use case are also available in the literature. Therefore, while the study shows great promise, the data preprocessing stage, such as missing value imputation, feature selection, etc., is very basic and could benefit from much more involvement. I would appreciate the authors to consider noting out to the reader in the future work section, for instance, that the missing value imputation technique employed is functional, but it is still uncertain if it is the most appropriate, and that future work could benefit from further exploration. By further exploration, here are two examples: one of which is a data-driven approach that selects (i.e., sort of in a brute force way) the most efficient missing value imputation technique for a longitudinal dataset, and the other is a Bayesian optimisation's system focusing on missing value imputation in general, not necessarily for longitudinal data only, for which I believe the search space could be tailored to include only methods applicable to longitudinal datasets. In the meantime, the first system is less complex in terms of its use of***

carried-forward and backward-based missing value imputations and supervised-based missing value imputation techniques, and it has been evaluated in a longitudinal classification setting. The second system is more complex because it uses Bayesian to seek out the most efficient candidate setting under a time constraint [1, 2].

We are appreciative of the reviewer's detailed comments and added evaluation of several imputation strategies. Our data consist of baseline variables and longitudinal features. The baseline variables include patient demographics, comorbidities, and other neurocognitive measurements. The comorbidities are binary variables, and we only included those with ≤ 7 missing values out of 1603 samples (missing rate ~ 0.44%), which is small enough to legitimately impute the missing values as 0 (no existence) since it is the major class for all comorbidities. For the remaining numerical baseline variables, we used two imputation strategies: (1) simple imputation with mean values of each column, which assumes the missing pattern to be MCAR; (2) multiple imputation by chained equation (MICE), which assumes the missing pattern to be MAR. For longitudinal variables, the missing values are mainly certain observations for some time steps in each patient. We further compared three imputation methods: (1) last observation carried forward (LOCF), assuming that the longitudinal data has temporal stability as the cohorts we used are placebo arms of clinical trials; (2) linear imputation, which assumes temporal linearity between observed time steps; (3) MICE, which assumes the missing pattern to be MAR and depend on other observed values. For longitudinal imputation (2) and (3), we avoided the temporal data leakage by imputing data within the observed periods according to the specific observation periods for each model. Thus, we have 6 combinations of imputation methods between baseline and longitudinal variables. The 6 imputation strategies were evaluated by the internal split and 10-fold cross-validation. The AUROC and AUPRC plots are reported in the appendix. We found that the two imputation strategies for baseline variables do not show significant difference, while the MICE imputation for longitudinal variables showed marginal improvement. As a result, we updated our imputation strategies by using MICE for both baseline and longitudinal variables.

We reported our experiments on imputation evaluation in the supporting information S1 Appendix "Imputation strategies" section:

> **Imputation strategies**
>
> We tried several imputation strategies to deal with missing data in our baseline variables and longitudinal variables. The baseline variables include patient demographics, comorbidities, and other neurocognitive measurements. The comorbidities are binary variables, and we only included those with ≤ 7 missing values out of 1603 samples (missing rate ~ 0.44%), which is small enough to legitimately impute the missing values as 0 (no existence) since it is the major class for all comorbidities. Then, for the remaining numerical baseline variables, we used two imputation strategies: (1) simple imputation with mean values of each column, which assumes the missing to be MCAR; (2) multiple imputation by chained equation (MICE), which assumes the missing to be MAR. For longitudinal variables, the missing values are mainly certain observations for some time steps in each patient. We further compared three imputation methods: (1) last observation carried forward (LOCF), assuming that the longitudinal data has temporal stability as the cohorts we used are placebo arms of clinical trials; (2) linear imputation, which assumes temporal linearity between observed time steps; (3) MICE, which assumes the missing to be MAR and depend on other observed values. For longitudinal

imputation (2) and (3), we carefully avoided the temporal data leakage by imputing data within the observed periods according to the specific observation periods for each model. Thus, we have 6 combinations of imputation methods between baseline and longitudinal variables. The 6 imputation strategies were evaluated by the internal split and 10-fold cross-validation. The AUROC and AUPRC plots are reported in the appendix. We found that the two imputation strategies for baseline variables do not show significant difference, while the MICE imputation for longitudinal variables showed marginal improvement. As a result, we updated our imputation strategies by using MICE for both baseline and longitudinal variables.

We also added S2 Fig to show AUROCs and AUPRCs of RP prediction models of different imputation strategies:

S2 Fig. AUROCs and AUPRCs of RP prediction models of different imputation strategies.

(A) Baseline variables: mean imputation. Longitudinal variables: 1) last observation carried forward (LOCF); 2) linear imputation; and, 3) multiple imputation by chained equation (MICE).
(B) Baseline variables: MICE. Longitudinal variables: 1) LOCF; 2) linear imputation; and, 3) MICE.
(C) Baseline variables: 1) mean imputation; and, 2) MICE. Longitudinal variables: MICE.

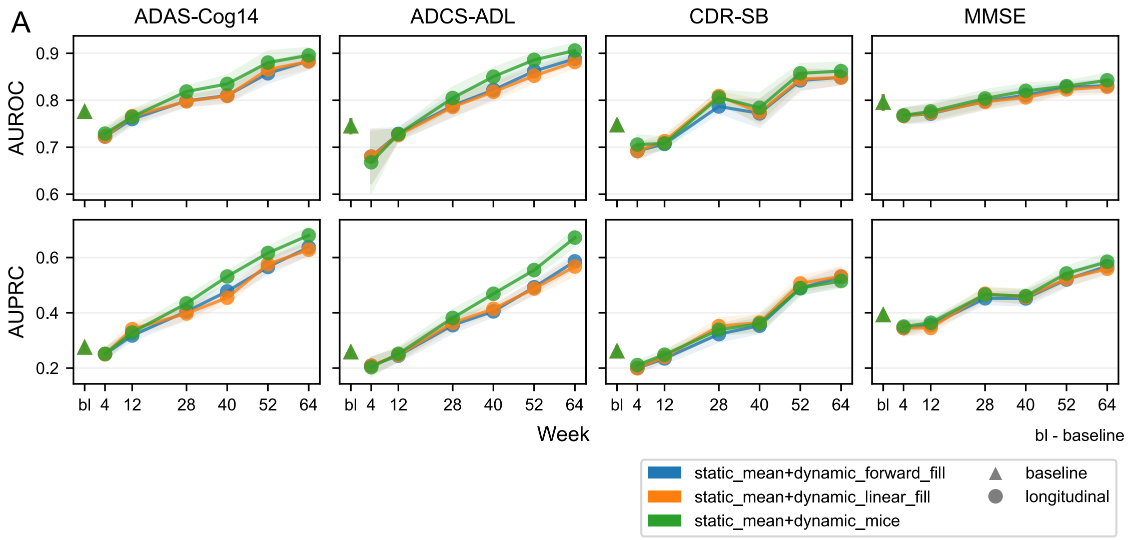

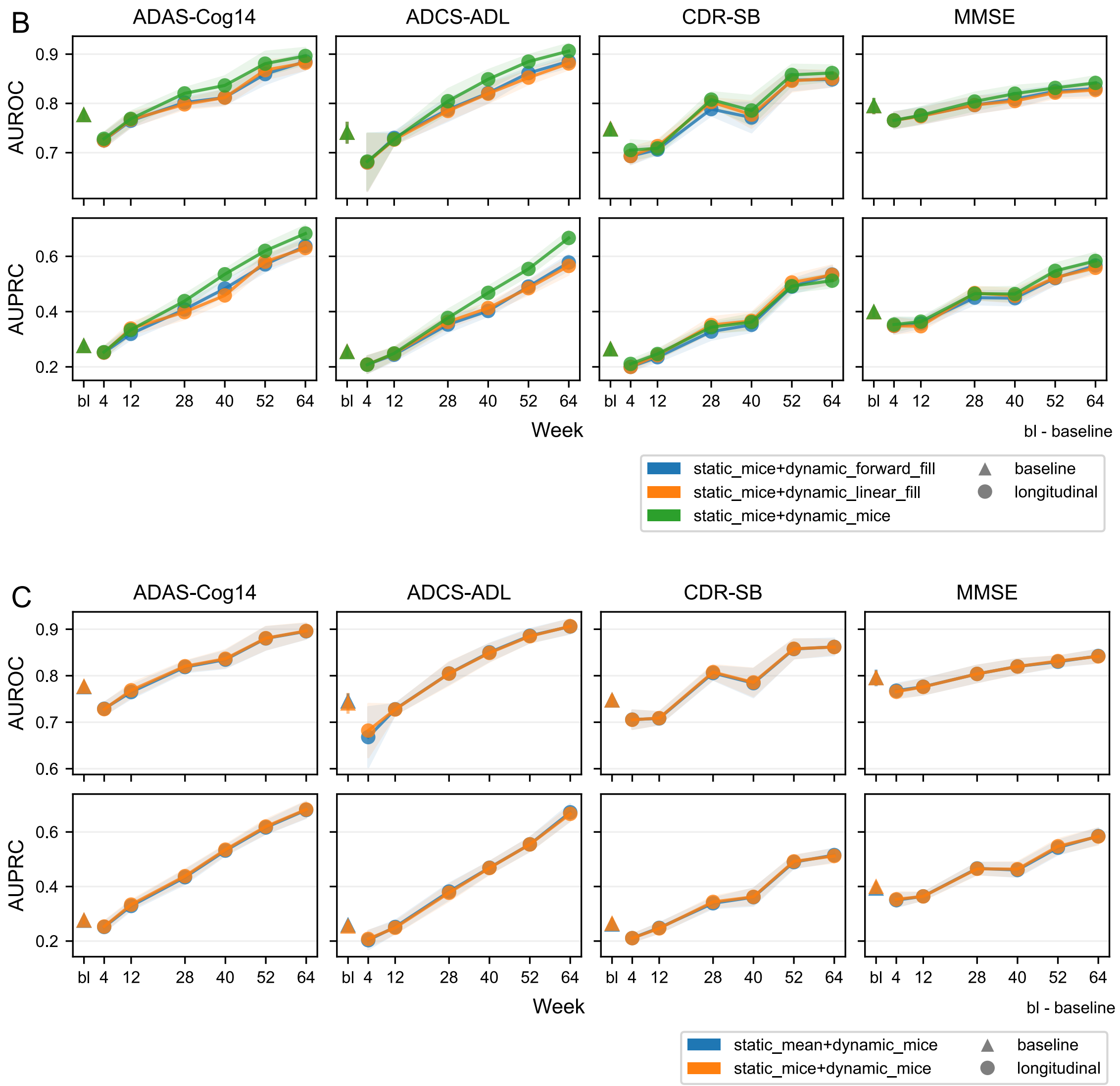

In the main text, the imputation part of “Data preprocessing” section in “Materials and Methods” now reads:

> Missing values in longitudinal variables were also imputed by MICE, assuming missing at random. We carefully avoided the temporal data leakage by imputing data within the observed periods according to the specific observation periods for each model. S1 Appendix and S2 Fig show details regarding how we evaluate and select from several imputation strategies.

We pointed out potential future works on data imputation to improve our models in the “Discussion” section by adding this:

> More imputation methods could be explored on our baseline and longitudinal data, such as the data-driven missing value imputation approach [39], Hyperimpute [40], or learning the data imputation during training by integrating the imputation module into the models.

The citations here are:

39. Ribeiro C, Freitas AA. A data-driven missing value imputation approach for longitudinal datasets. Artificial Intelligence Review. 2021;54: 6277–6307.

40. Jarrett D, Cebere BC, Liu T, Curth A, van der Schaar M. HyperImpute: Generalized Iterative Imputation with Automatic Model Selection. In: Chaudhuri K, Jegelka S, Song L, Szepesvari C, Niu G, Sabato S, editors. Proceedings of the 39th International Conference on Machine Learning. PMLR; 17--23 Jul 2022. pp. 9916–9937.

5. ***Comparing Adapted Supervised Machine Learning Algorithms and Neural Networks: While neural networks (NNs) and the attention mechanism offer a very promising approach to coping with longitudinal data classification, there are still significant challenges to be addressed. Adapted supervised machine learning algorithms appear to have emerged in the literature more recently [3, 4]. The overarching concept is to modify a standard machine learning algorithm, such as a decision tree, random forest, XGBoost, etc., to specifically address the temporality challenge that longitudinal data presents. As a result, while the study shows great promise, it would be advantageous to note that further exploration between NNs and adapted machine learning algorithms for longitudinal classification could be conducted with the goal of determining which of the two types of models is realistically more efficient for this medical use case, and even further, in a multi-objective analysis, which of the two models outputs the best trade-off between, e.g., accuracy and interpretability. Consequently, I would appreciate it if the authors would consider pointing out to the reader, for instance, in the section on future work, that the investigation of adapted machine learning algorithms for longitudinal classification could be a worthwhile research direction, given the many interpretability and medical context-related benefits that such models can also provide.***

We appreciate the reviewer's great suggestion and ideas for future work that could improve our research. Particularly, your suggestions on achieving the trade-off between accuracy and interpretability (for better accessibility) resonate with us, and our team indeed plans to "compress" the NN models with those suggested adapted algorithms. To address your suggestion, we explicitly pointed this out in the "Discussion" section by adding:

> Investigation could be conducted by using adapted supervised machine learning algorithms [41,42] and comparing them to our neural network models in terms of performance and efficiency for longitudinal classification.

The citations here are:

41. Ovchinnik S, Otero F, Freitas AA. Nested trees for longitudinal classification. Proceedings of the 37th ACM/SIGAPP Symposium on Applied Computing. New York, NY, USA: Association for Computing Machinery; 2022. pp. 441–444.

42. Ribeiro C, Freitas A. A New Random Forest Method for Longitudinal Data Classification Using a Lexicographic Bi-Objective Approach. 2020 IEEE Symposium Series on Computational Intelligence (SSCI). 2020. pp. 806–813.

6. ***While the study demonstrates promise, we would like to emphasize the importance of accelerating research in this direction via reproducibility. Perhaps you are restricted by your funding or the data provider, but code reproducibility is a very promising first step, even though the data needs licensing and an external request by the interested party. Consequently, I would like the author to consider adding either to the appendix or to the subsections on data availability, contributors, etc., an additional section pertaining to offering the reader a potential open-source hosted code of your architecture and any scripts of your experiments, allowing any interested reader to begin with concrete as opposed to starting from scratch. If you are not permitted or do not have a hosted-based service with your code, such as GitHub, GitLab, etc., for your research, I would kindly understand that it could take too long to produce such a thing and that, at this stage, this is not a significant point to consider. Despite this, the research community greatly values codes and how to reproduce papers' experiments, such as with scripts; therefore, we highly recommend kindly considering this in the future.***

   We thank the reviewer's suggestion and agree that it would be beneficial to the research community to make the code open source. We published the code of our models, training process, and evaluation on the GitHub repo (https://github.com/xiaotian0328/AD-Rapid-Progression-Prediction). We also reported the specific frameworks and libraries we used in S5 Table.

   Now, the "Data Availability" section reads:

   > Data access restrictions apply. Interested parties may contact Eli Lilly and Company for dataset licensing (https://www.lilly.com/clinical-research/clinical-trials) and request the data following the step-by-step process on the Vivli Platform (https://vivli.org/ourmember/lilly/). Code for building, training, and evaluating the models is available on the GitHub repo (https://github.com/xiaotian0328/AD-Rapid-Progression-Prediction). Specific frameworks and libraries we used are reported in S5 Table.

   We added this sentence in the "Overview" section in "Materials and Methods" to guide the readers to this:

   > The code for building, training, and evaluating the models can be found on GitHub (https://github.com/xiaotian0328/AD-Rapid-Progression-Prediction).

   We added Table S5 in supporting information reporting the frameworks and libraries for model building, training, and evaluation.

   S5 Table. Frameworks and libraries used for building, training, and evaluating the models.

GPU	NVIDIA V100
Container image	NVIDIA PyTorch container image 22.12
CUDA	11.8.0
Python	3.8.10
PyTorch	1.14.0a0+410ce96
Scikit-learn	1.3.2
NumPy	1.22.2
Pandas	1.5.2
Matplotlib	3.7.3

Captum	0.6.0

V. Conclusion

In conclusion, we sincerely appreciate the insightful feedback provided by the reviewers, which has significantly contributed to enhancing the quality and robustness of our study. We have diligently addressed each comment, making comprehensive revisions to our manuscript to reflect these valuable suggestions. Our responses include detailed explanations for the changes implemented, and we believe these modifications have notably strengthened the methodology and overall clarity of the paper. We are hopeful that our revisions meet the approval of the reviewers and align well with the high standards of PLOS Digital Health. We are grateful for the opportunity to improve our work through this rigorous review process and thank the reviewers and editors for their time and constructive critiques.
