# Supplementary material for: Deep learning to predict rapid progression of Alzheimer’s disease from pooled clinical trials: A retrospective study
Source: PLOS Digit Health. 2024 Apr 10;3(4):e0000479. doi: 10.1371/journal.pdig.0000479 (PMC11006164; doi:10.1371/journal.pdig.0000479)
Supplement: Attachment — Submitted filename: Response Letter - Plos Digital Health Revision 2.docx [file pdig.0000479.s014.docx]

Dear Editor and Reviewers,

We are grateful for your valuable feedback and reviews, which helped us significantly improve the quality of the paper. In this letter, we will respond to the new questions raised by the reviewers in detail.

First, we express our sincere gratitude for Review #1 and Review #4 for your endorsement of our revision. Your great suggestions improved our manuscript a lot and made it more robust and polished.

Also, we appreciated the further comments from the journal and Review #3. Below is the point-to-point response to all comments.

**I. Journal Requirements**

1. ***Please review your reference list to ensure that it is complete and correct. If you have cited papers that have been retracted, please include the rationale for doing so in the manuscript text, or remove these references and replace them with relevant current references. Any changes to the reference list should be mentioned in the rebuttal letter that accompanies your revised manuscript. If you need to cite a retracted article, indicate the article's retracted status in the References list and also include a citation and full reference for the retraction notice.***

Thank you for your reminder. We have checked all the references cited in our manuscript and ensured that they are complete and corrected, and none of them have been retracted.

2. ***Please amend your detailed Financial Disclosure statement. This is published with the article. It must therefore be completed in full sentences and contain the exact wording you wish to be published.***

***a. State the initials, alongside each funding source, of each author to receive each grant.***

***b. State what role the funders took in the study. If the funders had no role in your study, please state: “The funders had no role in study design, data collection and analysis, decision to publish, or preparation of the manuscript.”***

***c. If any authors received a salary from any of your funders, please state which authors and which funders.***

***If you did not receive any funding for this study, please simply state: “The authors received no specific funding for this work.”***

Thank you for clarifying the Financial Disclosure details. Now, the “Funding” section is as follows:

PS is funded by the McCord Family Professorship in Neurology, the Umphrey Family Professorship in Neurodegenerative Disorders, multiple NIH grants, several foundation grants, and contracts with multiple pharmaceutical companies related to the performance of clinical trials. He serves as a consultant and/or speaker for Eli Lilly, Eisai, and Acadia Pharmaceuticals. There is no perceived financial conflict between these activities and this manuscript. XJ is CPRIT Scholar in Cancer Research (RR180012), and he was supported in part by Christopher Sarofim Family Professorship, UT Stars award, UTHealth startup, the National Institute of Health (NIH) under award number R01AG066749, R01LM013712, R01LM014520, R01AG082721, R01AG066749, U01AG079847, U01TR002062, U01CA274576, and the National Science Foundation (NSF) #2124789.

**II. Response to Reviewer 3**

1. ***Small frequency issue: American Indian or Alaska Native, multiple race, APOE type E2/E2 have 1, 4, and 2 frequencies, respectively. Any coefficients for these categories are not interpretable, particularly since the authors used cross-validation to build the model. It was suggested to merge small frequency cells to avoid model convergence issues.***

We understand your concern, and thank you for pointing this out. We did not include “American Indian or Alaska Native” or “Multiple Races” during our model training, but we did include “APOE E2/E2”, which only has 2 positive samples in our dataset (S1 Table). However, since this is only one of the 151 variables used in our deep-learning models, it would not noticeably influence our models’ behavior. Our models were robust and did not show convergence issues during training. Nonetheless, we appreciate your comments and will consider this advice in our future works.

2. ***Reporting guideline: Using a checklist/guideline for reporting the study was suggested. Examples include TRIPOD (doi: 10.1186/s12916-014-0241-z), TRIPOD-AI (doi: 10.1136/bmjopen-2020-048008)***

We thank you for this suggestion. Using a checklist to report our study can make our contributions clearer. We have added the S6 Table in the supporting information to report the TRIPOD checklist about our paper:

**S6 Table. TRIPOD checklist of this study.**

| Section/topic | Item | Development or validation? | Checklist item | Section/Paragraph |
| --- | --- | --- | --- | --- |
| Title and abstract |  |  |  |  |
| Title | 1 | D;V | Identify the study as developing and/or validating a multivariable prediction model, the target population, and the outcome to be predicted. | Title |
| Abstract | 2 | D;V | Summarizes objectives, study design, setting, participants, sample size, predictors, outcome, statistical analysis, results, and conclusions. | “Abstract” and “Summary” sections |
| Introduction |  |  |  |  |
| Background and objectives | 3a | D;V | Explain the medical context (including whether diagnostic or prognostic) and rationale for developing or validating the multivariable prediction model, including references to existing models. | Paragraphs 1–3 in the “Introduction” section |
|  | 3b | D;V | Specify the objectives, including whether the study describes the development or validation of the model or both. | Paragraph 4 in the “Introduction” section |
| Methods |  |  |  |  |
| Source of data | 4a | D;V | Describe the study design or data source (e.g., randomized trial, cohort, or registry data), separately for the development and validation data sets, if applicable. | “Data source” subsection in the “Materials and Methods” section |
|  | 4b | D;V | Specify the key study dates, including start of accrual; end of accrual; and, if applicable, end of follow-up. | “Data source” subsection in the “Materials and Methods” section |
| Participants | 5a | D;V | Specify key elements of the study setting (e.g., primary care, secondary care, general population) including number and location of centers. | N/A |
|  | 5b | D;V | Describe eligibility criteria for participants. | “Data source” subsection in the “Materials and Methods” section |
|  | 5c | D;V | Give details of treatments received, if relevant. | N/A |
| Outcome | 6a | D;V | Clearly define the outcome predicted by the prediction model, including how and when assessed. | “Definitions of a rapid progressor” subsection in the “Materials and Methods” section |
|  | 6b | D;V | Report any actions to blind assessment of the outcome to be predicted. | N/A |
| Predictors | 7a | D;V | Clearly define all predictors used in developing the multivariable prediction model, including how and when they were measured. | “Variables” subsection in the “Materials and Methods” section |
|  | 7b | D;V | Report any actions to blind assessment of predictors for the outcome and other predictors. | N/A |
| Sample size | 8 | D;V | Explain how the study size arrived. | “Data processing” subsection in the “Materials and Methods” section |
| Missing data | 9 | D;V | Describe how missing data were handled (e.g., complete-case analysis, single imputation, multiple imputation) with details of any imputation method. | “Data processing” subsection in the “Materials and Methods” section |
| Statistical analysis methods | 10a | D | Describe how predictors were handled in the analyses. | “Data processing” subsection in the “Materials and Methods” section |
|  | 10b | D | Specify the type of model, all model-building procedures (including any predictor selection), and method for internal validation. | “Prediction model”, and “Evaluation” subsections in the “Materials and Methods” section |
|  | 10c | V | For validation, describe how the predictions were calculated. | “Evaluation” subsection in the “Materials and Methods” section |
|  | 10d | D;V | Specify all measures used to assess model performance and, if relevant, to compare multiple models. | “Evaluation” subsection in the “Materials and Methods” section |
|  | 10e | V | Describe any model updating (e.g., recalibration) arising from the validation if done. | N/A |
| Risk groups | 11 | D;V | Provide details on how risk groups were created if done. | N/A |
| Development vs. validation | 12 | V | For validation, identify any differences from the development data in setting, eligibility criteria, outcome, and predictors. | “Evaluation” subsection in the “Materials and Methods” section |
| Results |  |  |  |  |
| Participants | 13a | D;V | Describe the flow of participants through the study, including the number of participants with and without the outcome and, if applicable, a summary of the follow-up time. A diagram may be helpful. | N/A |
|  | 13b | D;V | Describe the characteristics of the participants (basic demographics, clinical features, available predictors), including the number of participants with missing data for predictors and outcome. | “Rapid progressors” subsection in the “Results” section |
|  | 13c | V | For validation, show a comparison with the development data of the distribution of important variables (demographics, predictors and outcome). | “Data” and “Rapid progressors” subsections in the “Results” section |
| Model development | 14a | D | Specify the number of participants and outcome events in each analysis. | “Data” and “Rapid progressors” subsections in the “Results” section |
|  | 14b | D | If done, report the unadjusted association between each candidate predictor and outcome. | N/A |
| Model specification | 15a | D | Present the full prediction model to allow predictions for individuals (i.e., all regression coefficients, and model intercept or baseline survival at a given time point). | “Observation periods”, “Prediction model”, “Evaluation”, and “Interpretation” subsections in the “Materials and Methods” section |
|  | 15b | D | Explain how to use the prediction model. | “Prediction model” subsection in the “Materials and Methods” section |
| Model performance | 16 | D;V | Report performance measures (with CIs) for the prediction model. | “Rapid progressor prediction” subsection in the “Results” section. |
| Model updating | 17 | V | If done, report the results from any model updating (i.e., model specification, model performance). | N/A |
| Discussion |  |  |  |  |
| Limitations | 18 | D;V | Discuss any limitations of the study (such as nonrepresentative sample, few events per predictor, missing data). | Paragraphs 9–11 in the “Discussion” section |
| Interpretation | 19a | V | For validation, discuss the results with reference to performance in the development data, and any other validation data. | Paragraphs 2–6 in the “Discussion” section |
|  | 19b | D;V | Give an overall interpretation of the results, considering objectives, limitations, results from similar studies, and other relevant evidence. | Paragraph 1 in the “Discussion” section |
| Implications | 20 | D,V | Discuss the potential clinical use of the model and implications for future research. | Paragraphs 7–8 in the “Discussion” section |
| Other information | | |  |  |
| Supplementary information | 21 | D;V | Provide information about the availability of supplementary resources, such as study protocol, Web calculator, and data sets. | “Supporting Information” section |
| Funding | 22 | D;V | Give the source of funding and the role of the funders for the present study. | “Funding” section |

*Items relevant only to the development of a prediction model are denoted by D, items relating solely to validation of a prediction model are denoted by V, and items relating to both are denoted D;V.

3. ***Calibration: The authors did not evaluate the model in terms of calibration. In addition to the discrimination/accuracy, the authors should report the calibration measure(s). Otherwise, the models are not reliable to use in clinical practice. Calibration is highly important with a large number of predictors with small events per variable as such in this study. Poor calibration can make predictions misleading. (doi: 10.1093/eurheartj/ehu207; 10.1186/s12916-019-1466-7)***

Thank you very much for this constructive suggestion. To address your feedback, we evaluated our model in terms of calibration. We fitted a spline calibration method on the output probabilities from the corresponding validation set for each model and then used it to calibrate the output probabilities from the corresponding test set. Finally, we averaged the 10-fold calibrated probabilities on the test set for each setting (4 labels by 6 observation periods) and plotted the calibration curves (reported as S4 Fig). As a result, our models achieved good calibration results overall, while models trained by the label defined by MMSE had better calibration than other ones.

We added S4 Fig as an additional supplementary document:

**S4 Fig. Calibration curves for all calibrated models.**

ECE: Expected Calibration Error; BS: Brier Score.

We added a paragraph in the S1 Appendix to describe the calibration procedure:

**Model calibration**

We calibrated all the models by fitting the spline calibration on the model predictions from corresponding validation sets and the ground-truth labels. We then averaged the calibrated probabilities over the 10 folds to get the final predictions on the test sets and plotted the calibration curves (here we used 7 bins) for all the models (S4 Fig). We also reported the expected calibration errors (ECEs) and Brier scores (BSs) for all the curves. ECE and BS are metrics that evaluate model calibration. The ECE is calculated by averaging the bin-weighted absolute differences between accuracy and confidence:

$$\mathrm{ECE}=\sum_{m=1}^{M} \frac{\left| B_{m} \right|}{N}\left| \mathrm{acc}\left( B_{m} \right)-\mathrm{con}\left( B_{m} \right) \right|$$

where $N$ is the sample size, $M$ is the number of bins (here $M=7$), $\left| B_{m} \right|$ is the number of samples in the bin $m$, $\mathrm{acc}\left( B_{m} \right)$ is the accuracy within bin $m$, and $\mathrm{con}\left( B_{m} \right)$ is the confidence of bin $m$ (averaged probabilities predicted by the models). The BS is defined by the mean squared difference between the probabilities predicted by the models and the ground-truth binary labels:

$$\mathrm{BS}=\frac{1}{N}\sum_{n=1}^{N} (p_{n}-g_{n})^{2}$$

where $N$ is the sample size, $p_{n}$ is the predicted probability of sample $n$, and $g_{n}$ is the ground-truth label of sample $n$. We can summarize that our models achieved good calibration results overall, while models trained by the label defined by MMSE have better calibration than other ones.

We also mentioned it in the main text in the “Evaluation” subsection of the “Materials and Methods” section:

To further ensure that our probabilistic predictions well align with the actual outcome distributions and that our models are reliable in clinical usage, we calibrated the model predictions and reported the calibration method and results in S1 Appendix and S4 Fig.

4. ***Combining machine learning with multiple imputation: It was unclear how the authors combined their models or model performance measures with multiple imputed datasets. Doi: 10.1037/met0000478***

We thank you for this comment. Overall, we defined several training settings based on 7 different observation periods (including baselines), 4 labels, and 2 validation settings (internal and external), resulting in 56 training settings. For each setting, we further used 10-fold cross-validation. As a result, we had to train 560 models for our whole pipeline. Considering the computational cost, we only fitted a single MICE imputation and generated only one imputed dataset in each fold. However, when taking the 10 folds of each training setting into account, all the missing data are imputed 9 times in training folds. In the end, we obtained our final analysis results by averaging the results from the 10-fold cross-validated models on the corresponding hold-out test sets.

We mentioned this in the “Imputation strategies” section of S1 Appendix by adding one sentence:

…according to the specific observation periods for each model. Note that when using MICE, we only generated one imputed dataset for each training fold, considering the computational cost that we had several training settings and used 10-fold cross-validation. Thus, we have 6 combinations of imputation methods…
